# Transcript Profiling Identifies NAC-Domain Genes Involved in Regulating Wall Ingrowth Deposition in Phloem Parenchyma Transfer Cells of *Arabidopsis thaliana*

**DOI:** 10.3389/fpls.2018.00341

**Published:** 2018-03-15

**Authors:** Yuzhou Wu, Jiexi Hou, Fen Yu, Suong T. T. Nguyen, David W. McCurdy

**Affiliations:** ^1^Centre for Plant Science, School of Environmental and Life Sciences, University of Newcastle, Callaghan, NSW, Australia; ^2^Jiangxi Provincial Key Laboratory for Bamboo Germplasm Resources and Utilization, Jiangxi Agricultural University, Nanchang, China; ^3^Department of Biological Sciences, Faculty of Science, Nong Lam University, Ho Chi Minh City, Vietnam

**Keywords:** transfer cells, wall ingrowths, *Arabidopsis thaliana*, phloem parenchyma, RNA-Seq, transcription factors

## Abstract

Transfer cells (TCs) play important roles in facilitating enhanced rates of nutrient transport at key apoplasmic/symplasmic junctions along the nutrient acquisition and transport pathways in plants. TCs achieve this capacity by developing elaborate wall ingrowth networks which serve to increase plasma membrane surface area thus increasing the cell's surface area-to-volume ratio to achieve increased flux of nutrients across the plasma membrane. Phloem parenchyma (PP) cells of Arabidopsis leaf veins *trans*-differentiate to become PP TCs which likely function in a two-step phloem loading mechanism by facilitating unloading of photoassimilates into the apoplasm for subsequent energy-dependent uptake into the sieve element/companion cell (SE/CC) complex. We are using PP TCs in Arabidopsis as a genetic model to identify transcription factors involved in coordinating deposition of the wall ingrowth network. Confocal imaging of pseudo-Schiff propidium iodide-stained tissue revealed different profiles of temporal development of wall ingrowth deposition across maturing cotyledons and juvenile leaves, and a basipetal gradient of deposition across mature adult leaves. RNA-Seq analysis was undertaken to identify differentially expressed genes common to these three different profiles of wall ingrowth deposition. This analysis identified 68 transcription factors up-regulated two-fold or more in at least two of the three experimental comparisons, with six of these transcription factors belonging to Clade III of the NAC-domain family. Phenotypic analysis of these *NAC* genes using insertional mutants revealed significant reductions in levels of wall ingrowth deposition, particularly in a double mutant of *NAC056* and *NAC018*, as well as compromised sucrose-dependent root growth, indicating impaired capacity for phloem loading. Collectively, these results support the proposition that Clade III members of the NAC-domain family in Arabidopsis play important roles in regulating wall ingrowth deposition in PP TCs.

## Introduction

The term “transfer cells” was first used by Gunning and co-workers to describe specialized phloem cells in minor veins that develop elaborate ingrowths of wall material hypothesized to facilitate enhanced transport capacity for phloem translocation (Gunning et al., 1968). Subsequent research over many decades has recognized that transfer cells (TCs) are ubiquitous across all higher plant taxonomic groups including fungi (Gunning and Pate, 1974), indicating a common genetic basis for their development (Offler et al., 2003). The enlargement in surface area of plasma membrane in TCs enables enriched densities of nutrient transporter proteins such as H^+^-ATPases and sucrose transporters (Harrington et al., 1997), thus promoting the capacity of the plasma membrane of TCs to transport solutes across apoplasmic/symplasmic boundaries encountered along nutrient acquisition and transport pathways in plants (Offler et al., 2003; McCurdy et al., 2008; Andriunas et al., 2013; Arun Chinnappa et al., 2013).

In the process of phloem loading, which actively accumulates solutes into the sieve element/companion cell (SE/CC) complex against a concentration gradient, vascular cells adjacent to SEs usually *trans*-differentiate into TCs with extensive wall ingrowths (Haritatos et al., 2000; Arun Chinnappa et al., 2013). In minor veins of *Arabidopsis thaliana* (Arabidopsis), phloem parenchyma (PP) cells adjacent to cells of the SE/CC complex *trans-*differentiate to become PP TCs (Haritatos et al., 2000). This arrangement of wall ingrowths adjacent to cells of the SE/CC complex is assumed to enhance phloem loading whereby sucrose delivered symplastically to PP TCs is released into the apoplasm via a membrane transport step and subsequently taken up into cells of the SE/CC complex via active membrane transport involving a sucrose/H^+^ co-transporter (SUC2) localized to the plasma membrane of CCs (Gottwald et al., 2000; Haritatos et al., 2000; Amiard et al., 2007). Recently, members of the AtSWEET family, namely AtSWEET11 and 12, were identified as the sucrose facilitators responsible for the apoplasmic unloading of sucrose from PP TCs (Chen et al., 2012).

Most TCs *trans*-differentiate from existing cell types, and this process occurs across normal programed development of particular tissues and organs, or can occur in response to biotic or abiotic stresses, which is probably an indirect response to demand for solute transport (Offler et al., 2003). Since TC differentiation in different tissues and cell types occurs commonly in response to diverse external stresses, it is hypothesized that inductive signals caused by these stresses induce TC differentiation. While signaling pathways involving auxin, ethylene and reactive oxygen species (ROS) involved in inducing the *trans*-differentiation of TCs have been elucidated (Dibley et al., 2009; Zhou et al., 2010; Andriunas et al., 2011, 2012), little is known about the key regulatory genes that respond to these signals to initiate wall ingrowth deposition. A number of studies have analyzed the transcriptomes of endosperm TCs in barley (Thiel et al., 2008, 2012; Thiel, 2014) and maize (Xiong et al., 2011), and epidermal TCs in *Vicia faba* cotyledons (Dibley et al., 2009; Zhang et al., 2015), and while these studies have provided valuable insights into the transcriptional changes associated with wall ingrowth deposition generally, only the study by Arun-Chinnappa and McCurdy (2016) focused on identifying putative regulatory transcription factors. This study identified at least 43 transcription factors which were up-regulated in epidermal TCs of *V. faba* cotyledons across wall ingrowth deposition (Arun-Chinnappa and McCurdy, 2016). The relevance of these transcription factors could not be tested, however, due to the absence of a reliable transformation protocol in *V. faba*.

In this study, we have used RNA-Seq to identify transcription factors putatively associated with TC development in PP cells of Arabidopsis, exploiting the genetic advantages of this species to test causative relationships between gene expression and TC development. Confocal analysis (see Nguyen and McCurdy, 2015) established that wall ingrowth deposition occurs rapidly across 4–6 day windows in both young cotyledons and first-emerged juvenile leaves, and occurs in a distinct basipetal gradient in mature adult leaves. These observations enabled RNA-Seq to identify transcription factors commonly expressed in cotyledons and leaves across these developmental windows. Strikingly, this analysis identified, in addition to other transcription factors, a cohort of closely related Class III-2 and III-3 NAC-domain transcription factors that were commonly up-regulated across PP TC development in all three experimental comparisons. Analysis of wall ingrowth deposition in *nac056/nac018*, a double mutant of the Class III-2 *NAC056* and *NAC018* genes, and *nac055/019/072*, a triple mutant of three Class III-3 NACs, namely *NAC055, NAC019*, and *NAC072*, showed significant reductions in juvenile leaves 1 and 2, and in day 10 cotyledons. Furthermore, sucrose-dependent root growth was reduced in *nac056/018*, possibly due to disrupted phloem loading. These results support a conclusion that wall ingrowth deposition in PP TCs of Arabidopsis involves redundant activities of developmentally important transcription factors, in particular members of the NAC-domain family.

## Materials and methods

### Plant growth

Seeds of Arabidopsis (Col-0 as wild-type and mutant lines as indicated) were sown directly onto moistened potting mix (Debco Seed Raising Mix) in small square pots (85 mm^2^), with 24 pots held in a single tray. After stratification in darkness at 4°C for 3 days, each tray of pots were transferred to an Adaptis A1000 growth chamber [16/8 h (day/night), 22°C/18°C (day/night), 90–120 μmol photons m^−2^ s^−1^ at the surface of the seeds]. Plants were fertilized immediately upon removal from cold (day 0) by adding 500 mL of 0.4% (v/v) Ionic Grow Plant Nutrient (pH ~ 6.0; Growth Technology) into each tray and weekly thereafter. At day 5, excess seedlings were removed with fine tweezers to leave one seedling per pot. The plants were then watered by adding 500 mL water to each tray every 2 or 3 days except the day when plants were fertilized.

### Confocal microscopy, image acquisition and semi-quantitative assessment of wall ingrowth abundance in PP TCs in stained tissue

Cotyledons and different rosette leaves were collected either directly at the beginning of the light cycle for seedlings less than 2-w old, or for older plants, they were covered with aluminum foil for 6 h to overnight to reduce starch content. These organs were fixed, cleared and stained in pseudo-Schiff propidium iodide solution as described in Nguyen et al. (2017). Mounted tissues were first viewed using a Leica MZ FLIII dissecting microscope equipped with a Zeiss AxioCam camera with AxioVision 4.0 software to obtain images of cotyledon/leaf morphologies, including vein patterning. Confocal imaging of stained tissues was then performed using an Olympus FluoView FV1000 confocal microscope using the parameters as described in Nguyen and McCurdy (2015). Briefly, *z*-stack images of between 0.5 and 2.0 μm thickness, depending on the age and size of the tissue, were generated using 488 nm Argon-ion laser excitation with an Olympus oil-immersion objective (UPLSAPO, 60 X O, NA:1.35). One-way scanning was applied to produce images with pixel resolution of 1,024 × 1,024 at a speed of 4 μs per pixel. Images were imported into Olympus Fluoro Viewer FV10-ASW version 4.0 for subsequent semi-quantitative assessment of wall ingrowth abundance in PP TCs according to the classification and scoring system described in Nguyen et al. (2017). For each cotyledon and leaf, scores of between 0 and 8 were assigned for wall ingrowth deposition taken from multiple images recorded from at least six locations across each cotyledon and first leaves, with these locations nominally recorded as either apical or basal to derive mean scores for wall ingrowth deposition across these locations. The mean value of scores derived from replicate plants (*n* ≥ 3) was calculated to derive a semi-quantitative analysis of wall ingrowth deposition in PP TCs. Student *t*-tests or one-way ANOVA were applied where appropriate to determine the statistical difference between different samples.

### Transmission electron microscopy

Dissected pieces of leaves and cotyledons were fixed according to Amiard et al. (2005) with some modifications. Briefly, small pieces of tissues (~5 mm^2^) were fixed in 2% (w/v) glutaraldehyde and 2.5% (w/v) formaldehyde in 70 mM sodium cacodylate buffer (pH ~ 6.9) overnight at 4°C on a rotating wheel. A vacuum pump was used to ensure submergence of the tissue pieces. Fixed tissues were then washed 3 × 15 min in 70 mM sodium cacodylate buffer (pH ~ 6.9) at room temperature, followed by post-fixation in 1% (w/v) osmium tetroxide in 70 mM sodium cacodylate buffer (pH ~ 6.9) for 1 h at room temperature. Subsequently, tissues were dehydrated through an ethanol series before being infiltrated into LR-White resin and embedded in capsules at ~60–70°C for 24–48 h. Ultrathin sections were cut and stained with uranyl acetate/lead citrate following standard procedures and viewed using a JEOL 1200 EX II transmission electron microscope (TEM).

### RNA extraction, quality control, cDNA library preparation and RNA-sequencing

Entire cotyledons and juvenile leaves were harvested with sterile scissors and collected into RNase-free microfuge tubes, immediately snap-frozen in liquid nitrogen and stored at −80°C until RNA extraction. For each biological replicate (*n* = 3), a total of 30 and 20 pairs of cotyledons at days 5 and 10, respectively, and 10 and two pairs of Leaf 1 and 2 at day 10 and 16, respectively, were collected. Mature adult leaves (*n* = 3) were dissected to harvest the apical and basal thirds of the blade from the same Leaf 12 at day 31, with the main vein in the basal section of leaves removed. These samples were also snap frozen in liquid nitrogen and stored at −80°C. Total RNA was extracted from samples using the RNeasy Plant Mini Kit (Qiagen) following the manufacturer's instructions, including two rounds of on-column gDNA elimination using DNase I digestion (Qiagen). The yield and purity of the extracted RNA was determined by Nanodrop (Thermo Fisher Scientific). At least 2.5 μg of total RNA from a single biological replicate was aliquoted into at least four technical replicates, to ensure at least three biological replicates from each single tissue type were processed for RNA-Seq. Total RNA samples were assessed and sequencing was performed by the Australian Genome Research Facility (AGRF, Melbourne). RNA quality was assessed using an Agilent 2100 Bioanalyzer, and three biological replicates from each tissue type were used to construct cDNA sequencing libraries using the Illumina TruSeq stranded mRNA sample preparation protocol. Each sample was subjected to either 100 bp (cotyledons and juvenile leaves) or 50 bp (apical and basal thirds of adult leaves) single-end sequencing using the Illumina HiSeq-2000 platform.

### RNA-seq analysis

Sequencing of the samples from cotyledons, juvenile leaves and apical/basal third of adult leaves yielded an average of 40 M (cotyledons and juvenile leaves 1 and 2, 100 bp) and 54 M (apical/basal adult leaves, 50 bp) reads for each of the biological replicates, as analyzed by Illumina CASAVA pipeline version 1.8.2 (conducted by AGRF). Raw reads of all samples have been deposited in Gene Expression Omnibus (GEO, NCBI) under accession number GSE107778. Raw reads from each sample were reloaded and analyzed in CLC Genomics Workbench 8.0 (Qiagen) installed on an Intel® Xeon® workstation with 64 Gb RAM. The first 10 nucleotides from the 5′ end of each read was removed to reduce positional sequence bias, and reads with quality scores lower than 0.05 and with more than two ambiguous nucleotides were trimmed according to CLC procedures. Subsequently, adapter sequences were removed according to the Illumina Adapter List. RNA-Seq analysis was performed in CLC Genomics Workbench 8.0 by mapping the trimmed reads to the TAIR10 Arabidopsis reference genome sequence, under default settings. Reads Per Kilobase (of exon) per Million (RPKM) mapped reads were calculated as expression values describing expression per gene. Biological samples from the same tissue types were grouped and temporal and spatial comparisons were undertaken to identify differentially-expressed genes across the developmental time-points representing the three experimental comparisons (see Table 1). A series of quality control surveys, including box plots and Principal Component Analysis (PCA) were also performed according to CLC Genomics Workbench 8.0. Thereafter, statistical analysis identifying differentially expressed genes (DEGs) was conducted using the tool *Empirical analysis of DGE* in CLC Genomics Workbench. This tool implements the “Exact Test” for two-group comparisons as developed by Robinson and Smyth (2008) and incorporated in the EdgeR Bioconductor package (Robinson et al., 2010). More details of the analysis are described in the subsequent Results sections.

**Table 1:** Summary of total gene numbers, DEGs, transcription factors and related statistics derived from RNA-Seq analysis of the three experimental comparisons of wall ingrowth deposition in PP TCs.

**Experiment Comparison**	**(i) Cotyledon day 5 vs. day 10**		**(ii) First leaves day 10 vs. day 16**		**(iii) Leaf 12 (day 31) basal vs. apical**	
PP TC class^a^	I	IV	I	IV	I	III
Non-expressed^b^	14,491		14,157		14,259	
*P* < 0.05^c^	8,038		10,998		5,111	
Infinity FC^d^	+∞	−∞	+∞	−∞	+∞	−∞
	267	378	459	617	133	98
FC cut-off^e^	≥2	≤ −2	≥2	≤ −2	≥2	≤ −2
	1,092	1,654	2,209	2,623	529	706
Trimmed^f^	98	63	119	67	63	120
	**Up**	**Down**	**Up**	**Down**	**Up**	**Down**
DEGs^g^	1,261	1,969	2,549	3,173	599	684
Transcription factors^h^	72	153	168	232	64	71

a *PP TC class (I to V) defined by semi-quantitative assessment of wall ingrowth abundance. Class I—no wall ingrowths; Class V—highly abundant/massive deposition of wall ingrowths. See Nguyen et al. (2017) for details*.

b *Numbers of genes that were identified as not expressed in both samples from each experimental comparison*.

c *Numbers genes that were expressed in at least one of the two samples, and showed FDR corrected P < 0.05*.

d *Numbers of genes showing infinite fold-change as±∞, because of non-expressed value in one of the two samples*.

e *Numbers of genes below the corresponding fold-change cutoff, when comparing samples with abundant vs. no wall ingrowths*.

f *Numbers of genes excluded from analysis due to aberrant expression between experimental comparisons*.

g Numbers of differentially expressed genes (DEGs) (after trimming) derived from combining “infinity FC” and “FC cutoff.”

h *Numbers of differentially expressed transcription factors (defined by PlantTFDB) among the DEGs*.

### Phylogenetic analysis of transcription factors

Full-length amino acid sequences of selected transcription factors from Arabidopsis were obtained from PlantTFDB under the latest modified (July 21, 2017) version 4.0 (http://planttfdb.cbi.pku.edu.cn/index.php?sp=Ath; Jin et al., 2015, 2017). These sequences were aligned using webservers of Clustal Omega (http://www.ebi.ac.uk/Tools/msa/clustalo/; Sievers et al., 2011). The aligned sequences were input into the protein maximum likelihood (proml) program in PHYLIP (PHYlogeny Inference Package version 3.69; Felsenstein, 1989) to construct phylograms which were then drawn by FigTree v1.4.2.

### Phenotypic analysis of T-DNA insertional mutants for selected NAC transcription factors

The double mutant *nac056/018* and the triple mutant *nac019/055/072* were kindly provided by Prof I. Hara-Nishimura (Kunieda et al., 2008) and Prof X. Dong (Zheng et al., 2012), respectively, and were confirmed in these publications to be homozygous knockout mutants for each respective gene. The *nac002/032* double mutant generated by the GABI-DUPLO project (Bolle et al., 2013) was obtained from NASC (Nottingham Arabidopsis Stock Centre). See Supplementary Table 4 for details of the mutant lines used in this study. The double homozygous status of *nac002/032* and effects on gene expression were determined by PCR genotyping and RT-PCR analyses, respectively, as described in Supplementary Material. Seeds of homozygous mutants and wild-type were germinated and grown in soil under uniform conditions as described earlier, and cotyledons and first pair of rosette leaves were collected at different time-points as specified. Procedures for fixation and staining as well as the methodologies involving confocal imaging and semi-quantitative assessment of wall ingrowth abundance in PP TCs in stained tissue were performed as described above.

### Root growth assay

Seeds of Col-0 and *nac056/018* were surface-sterilized and plated on 0.5x MS medium with or without 1% (w/v) sucrose, pH 5.8, and solidified with 0.5% (w/v) Phytagel. After stratification at 4°C for 3 d in darkness, plates were transferred to an Adaptis A1000 growth cabinet (conditions as described above under Plant Growth) and images recorded after 10, 13, and 20 days of vertical growth. Root length was measured from images using *ImageJ*.

## Results

### Temporal and spatial development of wall ingrowth deposition in PP TCs of cotyledons and leaves of arabidopsis

To provide a baseline determination of temporal and spatial development of PP TCs for RNA-Seq analysis, Arabidopsis plants were grown in soil under 16 h photoperiod conditions, and wall ingrowth deposition was assessed across cotyledon and leaf development. Representative images of Arabidopsis plants grown under these conditions from days 3 to 31 are shown in Supplementary Figure 1. Cotyledons first appeared at day 3 (Supplementary Figure 1A), and the first pair of rosette leaves emerged at day 7 (Supplementary Figure 1C), while Leaf 3 and 4 emerged at approximately ~day 12 (Supplementary Figure 1E). Leaves 5, 7, and 8 emerged at days 14, 17, and 19, respectively (Supplementary Figures 1F–H). Thereafter, rosette size increased substantially with a total of 10, 12, and 14 leaves by days 23, 25, and 31, respectively (Supplementary Figures 1I–K). Plants typically bolted by day 31 and developed short (<5 mm) inflorescence stems with associated cauline leaves and emerging flowers by this time (Supplementary Figure 1K).

To analyze temporal development of organ size under these growth conditions, images of seedlings were imported into *ImageJ* to measure cotyledon and first-pair (Leaf 1 and Leaf 2) surface areas (Supplementary Figure 2). Surface area for both cotyledons and leaves increased substantially in the early stages of growth, as seen by comparing days 3–10 for cotyledons and days 10–19 for leaves. This rate of expansion slowed, however, as organs aged and reached their fully expanded size by approximately day 17 for cotyledons and after day 25 for first leaves (Supplementary Figure 2).

**Figure 1:**
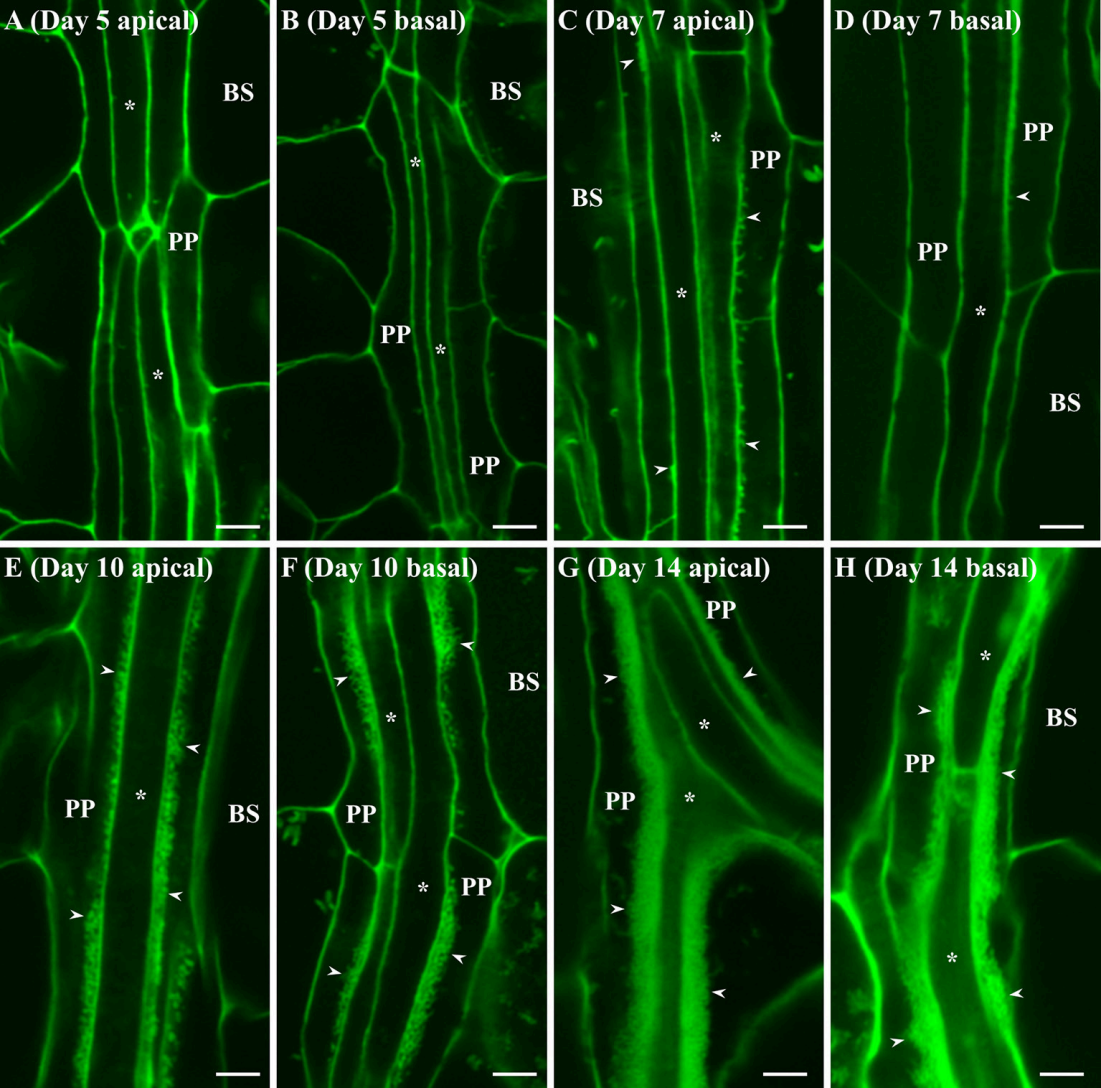
Confocal imaging of wall ingrowth deposition in PP TCs of Arabidopsis cotyledons. No evidence of wall ingrowth deposition was detected in PP cells of either the apical **(A)** or basal **(B)** half of cotyledons at day 5. By day 7, evidence of wall ingrowth deposition was detected as discrete fluorescent projections in PP TCs in the apical region of cotyledons **(C)** but only early stages of such protuberances were seen in the basal region **(D)**. By 10 days, PP TCs in both apical **(E)** and basal **(F)** regions of cotyledons showed extensive wall ingrowth deposition along the SE/CC border of PP TCs. At day 14, wall ingrowths in PP TCs in both apical **(G)** and basal **(H)** regions of the cotyledon were massively deposited and occupied a considerable volume of the PP TC. BS denotes bundle sheath cells; asterisks indicate SE/CCs; PP denotes PP cell or PP TC, as appropriate; arrowheads indicate wall ingrowths in PP TCs. The fluorescent fragments occasionally seen in BS cells correspond to remnant starch grains not completely extracted by the bleach treatment. Scale bars = 5 μm.

Cotyledons and leaves were harvested from these plants at different representative stages of growth and wall ingrowths in PP TCs were visualized by confocal microscopy of tissue stained with a modified pseudo-Schiff propidium iodide (mPS-PI) procedure (Nguyen and McCurdy, 2015). As shown in Figure 1, no wall ingrowths were detected in PP cells of leaf veins in either apical or basal regions of cotyledons at day 5 (Figures 1A,B). By day 7, small wall ingrowth protuberances were seen in PP TCs in apical regions of the cotyledon (Figure 1C), while only a few small protuberances were seen in basal regions (Figure 1D). By day 10, however, PP TCs in both apical and basal regions of the cotyledons showed abundant wall ingrowth deposition along the face of PP TCs adjacent to cells of the SE/CC complex (Figures 1E,F). By day 14, wall ingrowth deposition was highly abundant in PP TCs throughout the cotyledons, with wall ingrowths often occupying a considerable volume of each PP TC (Figures 1G,H). Similar massive levels of wall ingrowth deposition were seen in both apical and basal regions of cotyledons at day 17 (data not shown).

**Figure 2:**
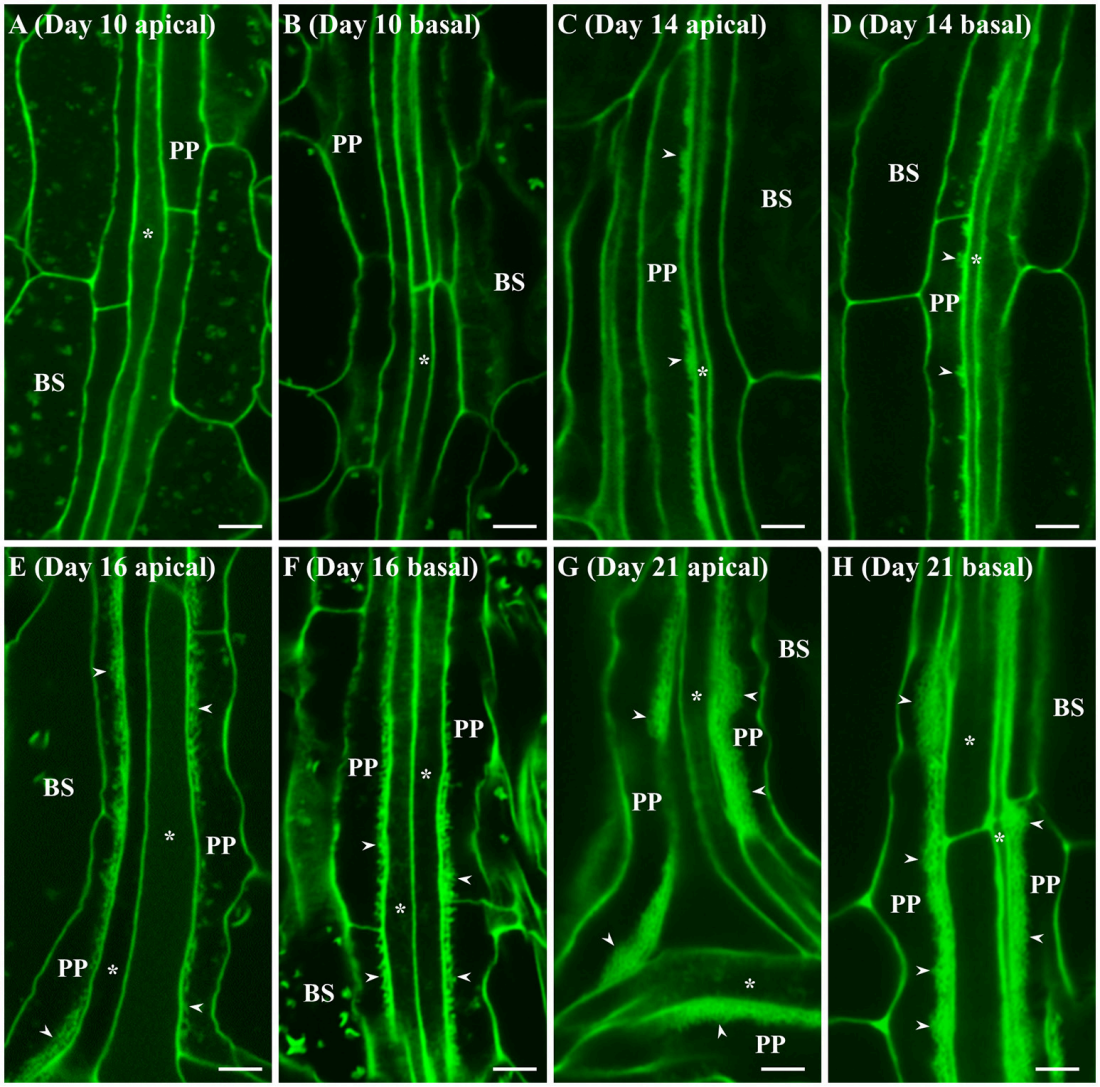
Confocal imaging of wall ingrowth deposition in PP TCs of Leaf 1 and 2 from Arabidopsis. At day 10, no wall ingrowths were detected in PP TCs in either apical **(A)** or basal **(B)** halves of the leaf. By day 14, the apical half of Leaf 1 and 2 **(C)** displayed dense clusters of wall ingrowths in PP TCs but this was less abundant in basal regions **(D)**. By day 16, continuous bands of wall ingrowth deposition were seen in PP TCs in both apical **(E)** and basal **(F)** regions of the leaf. At day 21, thick layers of wall ingrowth were deposited along the entire PP TC border in both apical **(G)** and basal **(H)** regions of the leaf. BS denotes bundle sheath cells; asterisks indicate SE/CCs; PP denotes PP cell or PP TC, as appropriate; arrowheads indicate wall ingrowths in PP TCs. The fluorescent fragments occasionally seen in BS and PP cells correspond to remnant starch grains not completely extracted by the bleach treatment. Scale bars = 5 μm.

A similar developmental profile was seen for wall ingrowth deposition in juvenile Leaf 1 and Leaf 2, albeit with different temporal characteristics. No wall ingrowths were seen in day 10 leaves in either apical or basal regions (Figures 2A,B). By day 14, however, substantial clusters of wall ingrowth deposition were seen in PP TCs in apical regions of these leaves (Figure 2C), but less so in basal regions (Figure 2D). By day 16, more extensive levels of wall ingrowth deposition were seen in PP TCs in both apical and basal regions of Leaf 1 or 2 (Figures 2E,F), and by day 21, highly abundant if not massive levels of deposition were seen in PP TCs throughout these leaves (Figures 2G,H). Similar massive deposition was also seen in both apical and basal regions of Leaf 1 and Leaf 2 at day 25 (data not shown).

**Figure 3:**
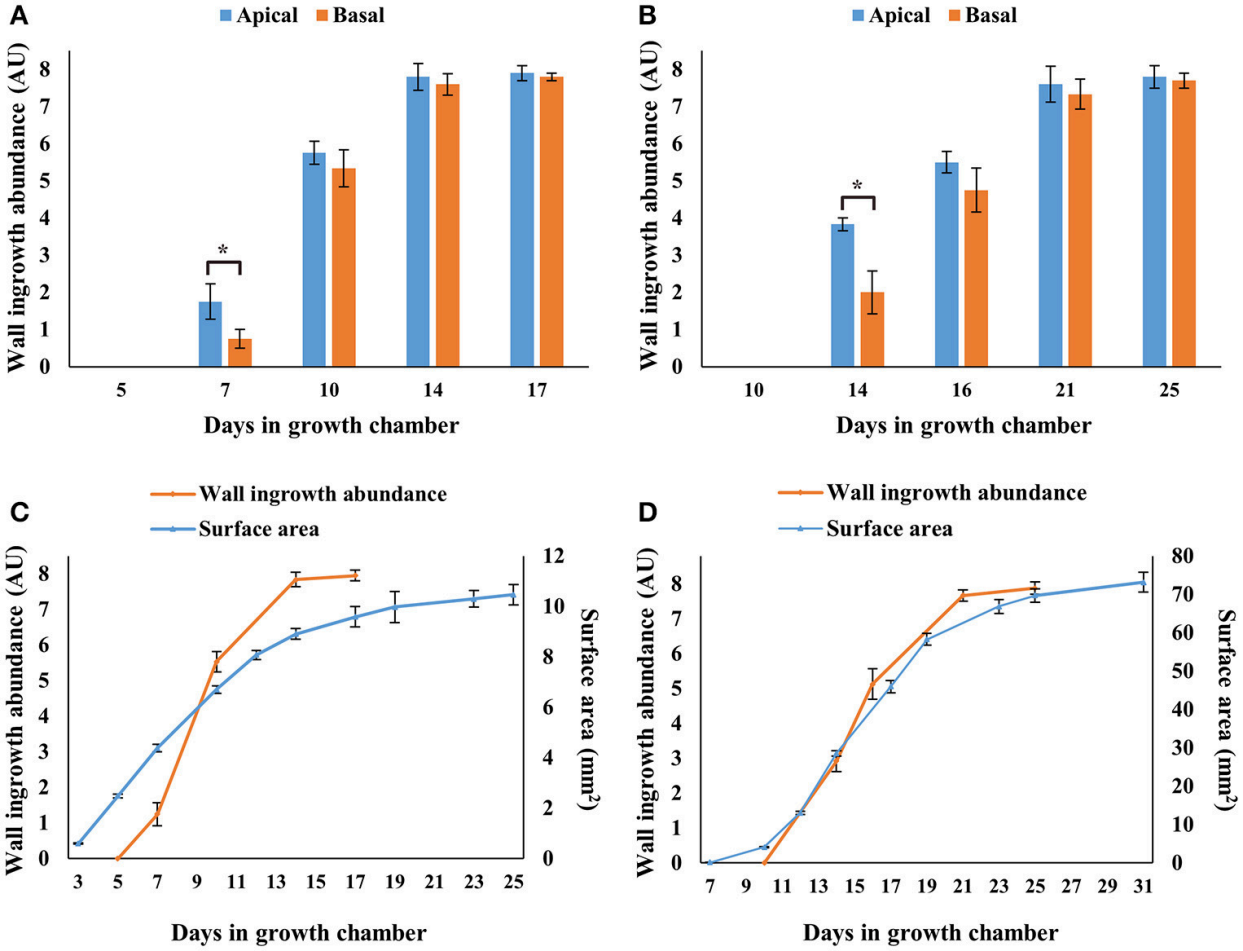
Semi-quantitative analysis of wall ingrowth deposition in cotyledons and first leaves at selected developmental stages, and relationship with surface area expansion across Arabidopsis rosette development. Wall ingrowth deposition was not detected in cotyledons **(A,C)** or Leaf 1 and 2 **(B,D)** at days 5 and 10, respectively, but increased dramatically thereafter and was maximally abundant by day 14 in cotyledons and day 21 in Leaf 1 and 2. Differences in wall ingrowth abundance in PP TCs between apical (blue bars) and basal (orange bars) regions of the organs was only statistically different in cotyledons at day 7 and leaves at day 14, where both showed ~50% reduction in the basal half compared to the apical half. Orange lines in **(C,D)** represent average scores of wall ingrowth abundance in PP TCs combined from apical and basal regions of cotyledons **(A)** and leaves **(B)**, respectively. Blue lines in **(C,D)** represent cotyledon and Leaf 1 and 2 surface areas, respectively, determined from images imported into *ImageJ*. Data shows mean ± SE of scores for wall ingrowth deposition in arbitrary units (see Nguyen et al., 2017), and for surface area in mm^2^. ^*^P < 0.05, *n* >3, student's *t*-test.

Semi-quantitative analysis of wall ingrowth deposition across apical and basal regions of developing cotyledons and Leaf 1 and 2 was undertaken as described in Nguyen et al. (2017). As shown in Figure 3, wall ingrowths were absent at days 5 and 10 of cotyledons and Leaf 1 and 2, respectively, but deposition was evident 2 days later in cotyledons (day 7; Figure 3A) and substantially present 4 days later in Leaf 1 and 2 (day 14; Figure 3B). Deposition of wall ingrowth material then progressed steadily in both organs to be highly abundant/massive (scores of >7 out of 8) by day 14 in cotyledons and by day 21 in Leaf 1 and 2 (Figures 3A,B). Similar levels of highly abundant wall ingrowths were seen in both apical and basal regions of cotyledons and Leaf 1 and 2 at day 17 and 25, respectively (Figures 3A,B). Despite differences in the abundance of wall ingrowth deposition between apical and basal regions in day 7 cotyledons and day 14 leaves, wall ingrowth abundance in PP TCs was uniform across these organs at all later stages examined (Figures 3A,B).

To provide semi-quantitative analysis of wall ingrowth deposition across developing organ size in both whole cotyledons and Leaf 1 and 2, apical/basal data sets for both organs were combined and plotted against organ surface area (Figures 3C,D). This analysis showed that wall ingrowth abundance increased in a roughly sigmoidal fashion in both cotyledons and Leaf 1 and 2 (Figures 3C,D), but the relationship between wall ingrowth abundance and Leaf 1 and 2 surface area was more closely matched temporally than that for cotyledons (Figures 3C,D). Nevertheless, the extent of wall ingrowth deposition plateaued by days 14 and 21 for cotyledons and first leaves, respectively, with both time-points being 3–4 days earlier than when these organs reached maximal size (day 17 for cotyledons and day 25 for first leaves).

**Figure 4:**
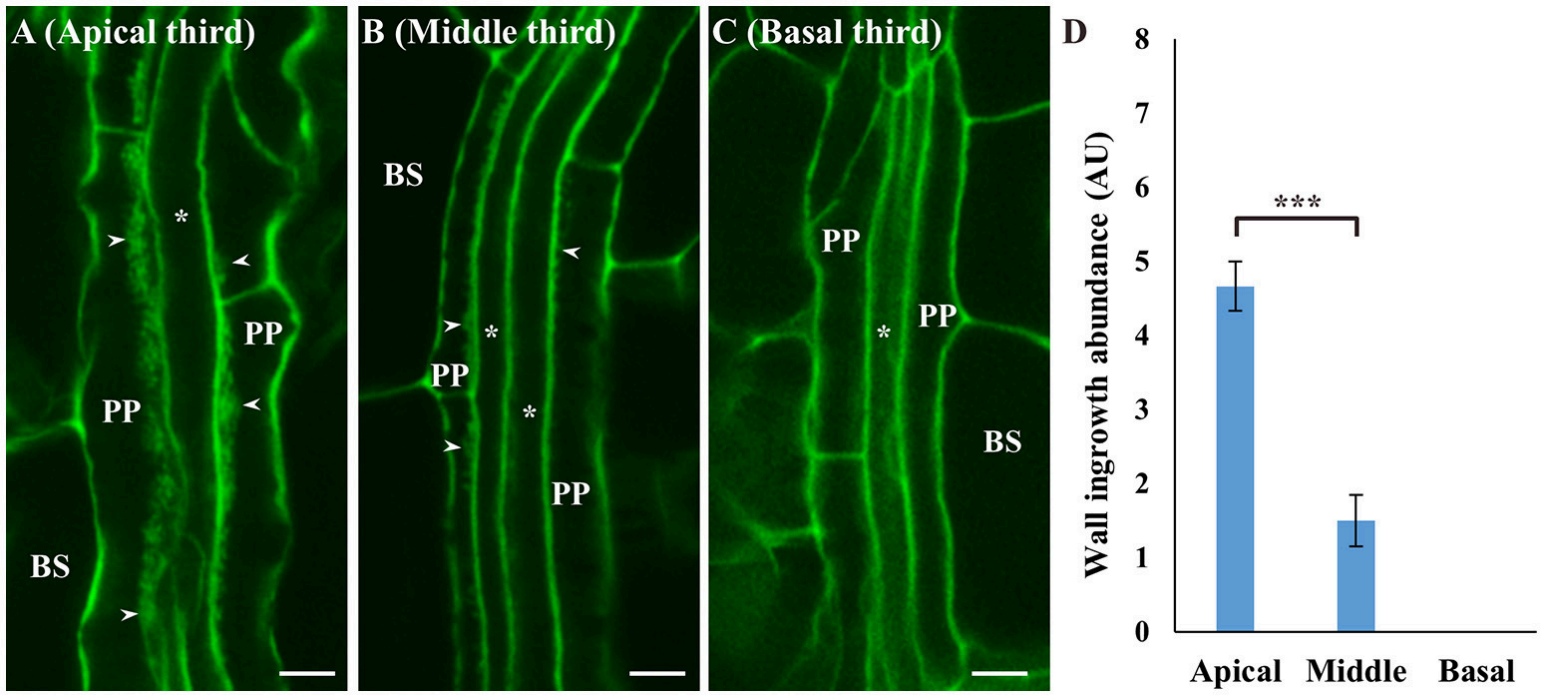
Deposition of wall ingrowths in PP TCs of minor veins in apical, middle and basal regions of rosette Leaf 12 at day 31. Thick stretches of wall ingrowth deposition can be seen in PP TCs in a minor vein in the apical third of the leaf **(A)**, with less abundant deposition in the middle third of the leaf **(B)**, and no wall ingrowths seen in PP cells in the basal third of the leaf **(C)**. BS denotes bundle sheath cells; asterisks indicate SE/CCs; PP denotes either PP or PP TCs as appropriate; arrowheads indicate wall ingrowths. Scale bars = 5 μm. **(D)** Semi-quantitative analysis of wall ingrowth deposition showed that wall ingrowth abundance significantly decreased from the tip (apical) to the base (basal) of the leaf. Data shows mean ± SE of scores for wall ingrowth deposition in arbitrary units. ^***^P < 0.001, student's *t*-test, *n* > 6).

Spatial development of ingrowth deposition was also analyzed in mature adult leaves. For this analysis, Leaf 12 from Arabidopsis plants at day 31 (see Supplementary Figure 1K) was stained using the mPS-PI procedure, and representative confocal images of wall ingrowths in PP TCs from the apical, middle and basal region of the leaf are shown in Figures 4A–C, along with semi-quantitative analysis of wall ingrowth deposition in these three regions (Figure 4D). Wall ingrowths were abundantly deposited as typically thick stretches or dense clusters of ingrowth material in PP TCs in the apical third of the leaf (Figure 4A), while less abundant and punctate patterns of deposition were seen in the middle region of the leaf (Figure 4B). No wall ingrowth material was detected in PP cells of minor veins at the base of the leaf (Figure 4C). This distinct basipetal gradient of ingrowth deposition was clearly evident from semi-quantitative analysis (Figure 4D). This analysis also showed that the abundance of wall ingrowth deposition in mature adult leaves (i.e., Leaf 12 from day 31 plants) was substantially reduced compared to the levels seen in mature cotyledons and juvenile leaves (Figure 3, and see Nguyen et al., 2017).

**Figure 5:**
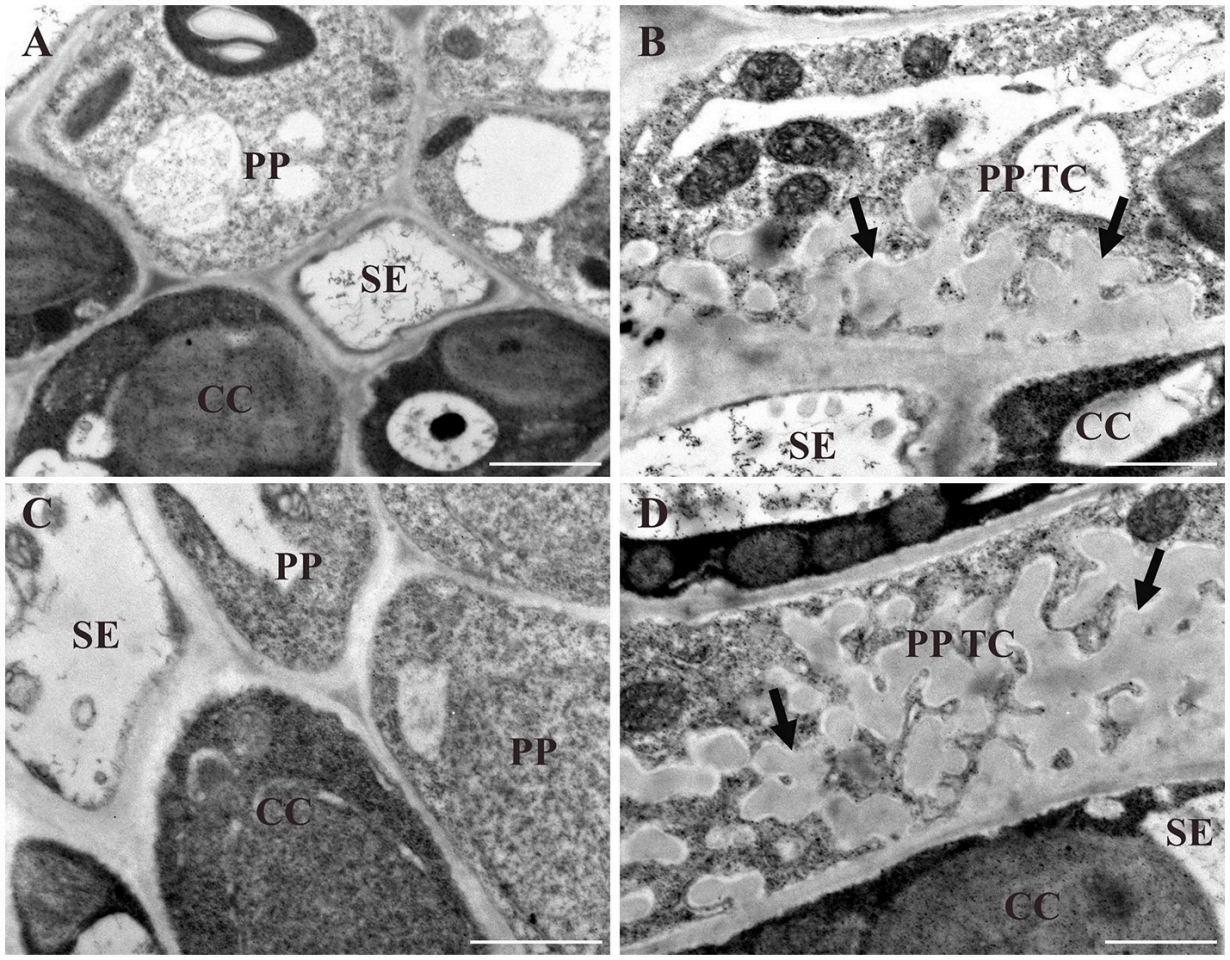
TEM analysis of different stages of wall ingrowth deposition in PP TCs. Wall ingrowths are absent in PP cells of day 5 cotyledons **(A)** or day 10 juvenile Leaf 1 **(C)**. By comparison, extensive deposition is seen in PP TCs in day 10 cotyledons **(B)** and day 16 Leaf 1 **(D)**. While these ingrowths occupy a substantial volume of the PP TC, their deposition is clearly restricted or focused to the face(s) of the PP TC abutting cells of the SE/CC complex. BS, CC and SE denote bundle sheath cell, companion cell and sieve element, respectively; PP denotes phloem parenchyma cell; PP TC denotes phloem parenchyma transfer cell; arrows indicate deposition of wall ingrowths. Scale bars = 1 μm.

To verify differences in wall ingrowth deposition as reported by confocal imaging of mPS-PI stained tissues, TEM of PP TCs in cotyledons and juvenile leaves was undertaken. Figure 5A shows the absence of wall ingrowths in PP cells of day 5 cotyledons, and highly abundant ingrowths in day 10 cotyledons (Figure 5B). Similarly, in juvenile Leaf 1, wall ingrowths were absent at day 10 (Figure 5C), but highly abundant by day 16 (Figure 5D). These results establish that confocal imaging of mPS-PI-stained tissue accurately reflects the levels of wall ingrowth deposition occurring in PP TCs.

Collectively, the observations reported in Figures 1–5 defined temporal differences in PP TC development in developing cotyledons and juvenile Leaf 1 and Leaf 2, as well as spatial differences in adult Leaf 12. These observations therefore provided three different scenarios for wall ingrowth deposition in PP TCs and thus an experimental platform for RNA-Seq to identify differentially expressed genes (DEGs) commonly up- or down-regulated across these scenarios, thus creating a pathway to identify regulatory genes putatively controlling wall ingrowth deposition in PP TCs.

### RNA-seq analysis of genes differentially expressed across PP TC development in developing cotyledons, juvenile leaves and mature adult leaves

Based on the profiles of wall ingrowth deposition shown in Figures 3, 4, RNA-Seq was performed on cotyledons harvested at days 5 and 10, juvenile Leaf 1 and 2 harvested at days 10 and 16, and the apical third and basal third (minus mid vein) of Leaf 12 at day 31. Collectively, this sampling enabled three different pair-wise comparisons for RNA-Seq analysis, namely: (i) cotyledons at days 5 vs. 10; (ii) Leaf 1 and Leaf 2 (first juvenile leaves) at days 10 vs. 16; and (iii) basal vs. apical thirds of Leaf 12 at day 31. Sample harvesting, RNA extraction and sequencing was performed as described in Materials and Methods. Trimmed reads of each sample contained at least 97% of the sequence with a Phred quality score of ≥Q30 (error probability ≤ 0.001), and a minimum of 98% of the trimmed reads from each sample were mapped to the Arabidopsis genome (TAIR10), of which at least 92% were uniquely mapped (see Supplementary Table 1 for mapping statistics as reported by CLC Genomics Workbench.

A summary of the temporal (i) and (ii) and spatial (iii) comparisons defined above, with corresponding categories of PP TC development, is presented in Table 1. Samples were analyzed statistically using the tool *Empirical analysis of DGE* (*Differential Gene Expression*) in CLC Genomics Workbench as described in Materials and Methods, with original total exon counts from each sample used as input. For each gene, if the average exon counts from all biological replicates of a sample were identified as <10, the gene was defined as not expressed in that sample (see Soneson and Delorenzi, 2013; Sha et al., 2015). Based on this condition, 14,491, 14,157 and 14,259 genes from Arabidopsis were identified as not expressed in each of the three experimental comparisons (i), (ii) and (iii), respectively, and these were consequently excluded from further analyses (Table 1). The numbers of DEGs expressed in at least one of the samples (exon counts ≥10) in each of the three comparisons are also summarized in Table 1, and described as follows: by defining the FDR-corrected *P*-value of below 0.05 as calculated by CLC Workbench, 8,038, 10,998, and 5,111 genes were identified in experimental comparisons (i), (ii), and (iii), respectively. In each of the comparisons between Sample A (e.g., day 5 cotyledons) vs. Sample B (e.g., day 10 cotyledons), fold-change (FC) was calculated based on Sample B relative to Sample A. These “relative abundance” values (normalized exon counts) were derived internally in the “Exact Test” algorithm, and depend on size of the samples, magnitude of the counts and estimated negative binomial dispersion, which in this case was a weighted combination of the tag-wise and common dispersion (CLC Genomics Workbench). For genes identified as not expressed in A but expressed in B (i.e., exon counts <10 in A but ≥10 in B), FC was defined as positive infinity (+∞); in contrast, for genes identified as expressed in A but not in B (i.e., exon counts ≥10 in A but <10 in B), FC was defined as negative infinity (−∞). In all other cases where genes were identified as expressed in both samples A and B (both exon counts ≥10), FC was calculated to be positive if Sample B was larger than Sample A, and negative if Sample A was larger than Sample B. Based on this analysis, 1,092, 2,209, and 529 genes showed at ≥2 FC in experimental comparisons (i), (ii), and (iii), respectively, while 1,654, 2,623, and 706 genes were down-regulated ≤2-fold in these three comparisons, respectively. Subsequently, genes showing ≥2-fold up-regulation for experimental comparisons (i), (ii), and (iii) were combined with genes showing positive ∞ FC to generate a list of genes defined as up-regulated for the experimental comparisons listed in Table 1. Lists of down-regulated genes were created similarly by combining genes showing significant negative FC with genes showing negative ∞ FC. Both lists were then trimmed by excluding genes that showed opposite or aberrant expression changes across the set of experimental comparisons. After this trimming, 1,261, 2,549, and 599 genes were identified as up-regulated across the three experimental comparisons, respectively, while 1,969, 3,173, and 684 genes were identified as commonly down-regulated across these comparisons (Table 1).

### Transcription factors differentially expressed across wall ingrowth deposition in PP TCs

**Figure 6:**
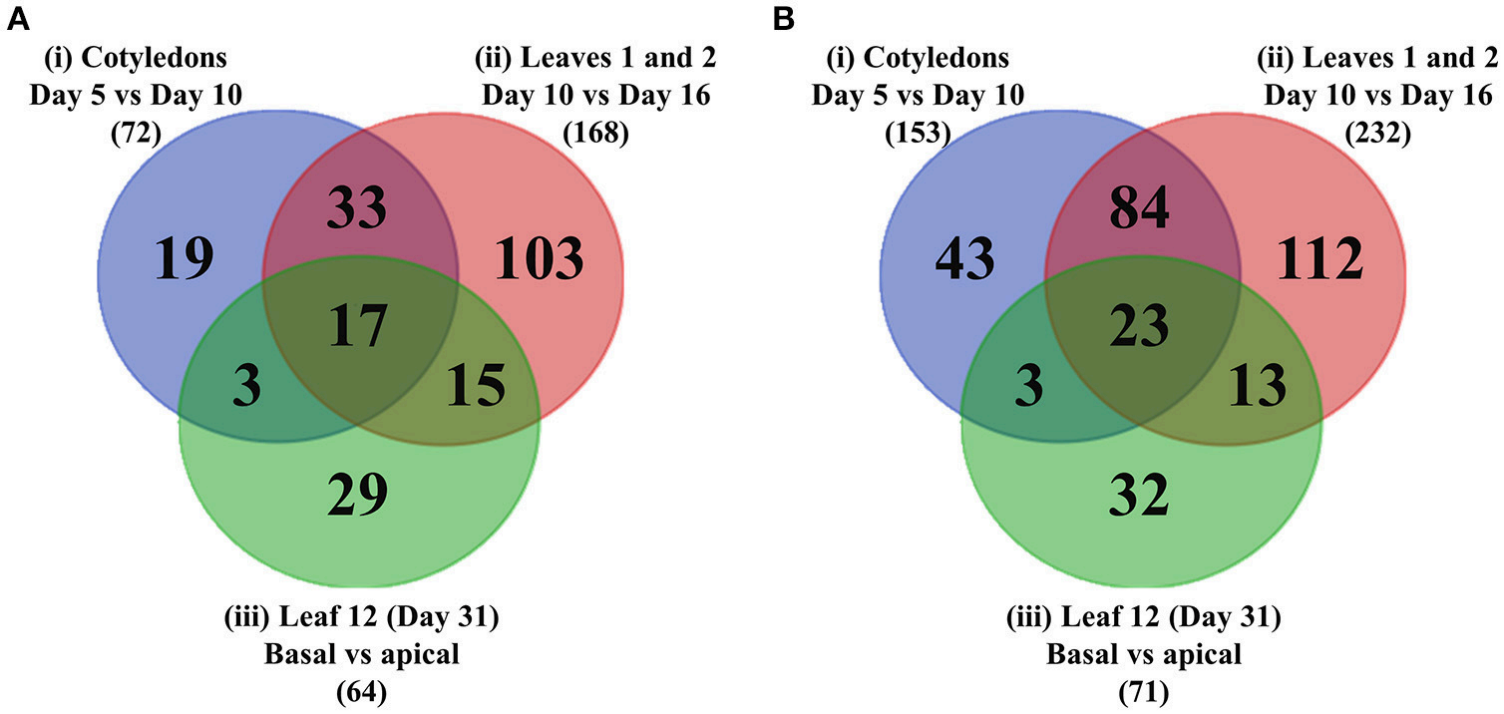
Venn diagrams showing intersections of transcription factors differentially up- or –down-regulated across wall ingrowth deposition in PP TCs of different organs from Arabidopsis grown. **(A)** Up-regulated genes. **(B)** Down-regulated genes. In each case the three circles represent the three experimental comparisons: Blue—cotyledons at day 5 vs. day 10; Red - first leaves at day 10 vs. day 16; Green - basal vs. apical thirds of Leaf 12 at day 31. Total number of differentially regulated genes in each of the three experimental comparisons, i.e., (i)–(iii), for each category is indicated in brackets.

Of the DEGs listed in Table 1, 72, 168, and 64 were identified as transcription factors (as defined by PlantTFDB) which were differentially up-regulated ≥2-fold for experimental conditions (i), (ii), and (iii), while 153, 232, and 71 transcription factors were identified as down-regulated across these criteria, respectively. To identify the intersections of these transcription factors between the different comparisons, a Venn diagram (Figure 6A) was generated which shows 219 unique transcription factors that were up-regulated in the three experimental conditions listed in Table 1. A similar Venn diagram for down-regulated genes is shown in Figure 6B. This analysis identified 68 transcription factors that were commonly up-regulated in at least two experimental comparisons, of which 17 of this cohort were commonly up-regulated across all three. These 17 transcription factors are listed in Table 2 according to aggregate fold-change across the three experimental conditions, while Supplementary Table 2 lists the remaining 51 transcription factors which are grouped into families and ranked according to highest individual FC score. Similarly, the 23 transcription factors that were commonly down-regulated in all three experimental comparisons are shown in Table 3, while the 100 that were down-regulated in two of the three comparisons are listed in Supplementary Table 3. Since the conceptual focus of this study, however, was to identify transcription factors that “switch on” the genetic network required for wall ingrowth biosynthesis, further consideration of down-regulated genes is restricted to the Discussion.

**Table 2:** Transcription factors (17 in total) showing differential up-regulation in all three experimental comparisons analyzing wall ingrowth deposition in PP TCs of Arabidopsis.

**Locus^a^**	**Symbol^b^**	**Family^c^**	**(i) Cotyledon day 5 vs. day 10**		**(ii) First leaves day 10 vs. day 16**		**(iii) Leaf 12 (day 31) Basal vs. apical**		**Aggregate FC score^g^**
			***P*^d^**	**FC^e^**	***P***	**FC**	***P***	**FC**	
AT3G15510	*NAC056*	NAC	0.00	11.2	0.00	62.1	0.00	4.2	77.5
AT5G15830	*AtbZIP3*	bZIP	0.00	14.6	0.00	40.1	0.00	2.4	57.1
AT1G69490	*NAC029*	NAC	0.00	6.5	0.00	10.1	0.00	3.9	20.5
AT1G52880	*NAC018*	NAC	0.00	5.6	0.00	10.8	0.00	2.9	19.3
AT1G75250	*RL6*	MYB-related	0.00	10.2	0.00	6.8	0.00	2.0	19.0
AT1G56010	*NAC021*	NAC	0.00	2.8	0.00	14.1	0.04	2.0	18.9
AT1G29600	*AT1G29600*	C3H	0.00	3.1	0.00	11.6	0.00	2.4	17.1
AT5G15800	*SEP1-2*	MIKC MADS	0.00	2.1	0.00	5.4	0.00	8.8	16.3
AT3G16770	*RAP2-3*	ERF	0.00	8.2	0.00	2.5	0.00	2.1	12.8
AT5G59780	*MYB59*	MYB	0.00	2.3	0.00	7.2	0.00	3.0	12.5
AT1G19510	*RL5*	MYB-related	0.00	3.1	0.00	2.8	0.00	3.1	9.0
AT4G32980	*ATH1*	TALE	0.00	3.8	0.00	2.6	0.00	2.2	8.6
AT1G69310	*WRKY57*	WRKY	0.00	2.2	0.00	3.6	0.00	2.1	7.9
AT2G40200	*BHLH51*	bHLH	0.00	2.0	0.00	3.4	0.04	2.4	7.8
AT1G02220	*NAC003*	NAC	0.00	2.2	0.00	2.3	0.00	2.5	7.0
AT1G27045	*ATHB-54*	HD-ZIP	0.00	+∞^f^	0.00	5.0	0.00	2.2	
AT3G26790	*FUS3*	B3	0.00	+∞	0.00	+∞	0.01	2.1	

a, b *Gene locus and symbols were retrieved from TAIR10*.

c *Transcription factors were classified into families based on the assignment rules in PlantTFDB*.

d *P-value adjusted by FDR, calculated by Empirical analysis of DGE in CLC Genomics Workbench*.

e *FC—fold-change in expression of genes between data sets (comparing samples with abundant vs. no wall ingrowths), calculated by Empirical analysis of DGE in CLC Genomics Workbench*.

f *+∞ indicates gene was not expressed (exon counts <10) in sample with no detectable wall ingrowths, but was expressed (exon counts≥10) in the comparison sample with abundant wall ingrowth deposition*.

g *Aggregate fold-change calculated for ranking purposes. Genes with +∞ fold change were not included in this calculation and are listed at the bottom of the table*.

**Table 3:** Transcription factors (23 in total) showing differential down-regulation in all three experimental comparisons analyzing wall ingrowth deposition in PP TCs of Arabidopsis.

**Locus^a^**	**Symbol^b^**	**Family^c^**	**(i) Cotyledon day 5 vs. day 10**		**(ii) First leaves day 10 vs. day 16**		**(iii) Leaf 12 (day 31) basal vs. apical**		**Aggregate FC score^g^**
			***P*^d^**	**FC^e^**	***P***	**FC**	***P***	**FC**	
AT4G00480	*BHLH12*	bHLH	0.00	−16.4	0.00	−17.9	0.00	−6.3	−40.6
AT2G02540	*ZHD3*	ZF-HD	0.00	−5.9	0.00	−12.9	0.00	−5.0	−23.8
AT4G38070	*BHLH131*	bHLH	0.00	−4.5	0.00	−16.6	0.00	−2.4	−23.5
AT4G16141	*AT4G16141*	GATA	0.00	−5.6	0.00	−10.5	0.00	−2.3	−18.4
AT4G23750	*CRF2*	ERF	0.00	−10.9	0.00	−5.5	0.00	−2.0	−18.4
AT3G57600	*DREB2F*	ERF	0.00	−7.7	0.00	−4.5	0.00	−2.9	−15.1
AT5G46880	*HDG5*	HD-ZIP	0.00	−3.4	0.00	−8.8	0.00	−2.0	−14.2
AT2G24645	*REM14*	B3	0.00	−4.7	0.00	−6.8	0.00	−2.1	−13.6
AT1G06180	*ATMYB13*	MYB	0.00	−4.7	0.00	−4.7	0.04	−2.3	−11.7
AT3G04850	*TCX4*	CPP	0.00	−3.7	0.00	−4.9	0.00	−2.0	−10.6
AT3G50870	*GATA18*	GATA	0.00	−2.6	0.00	−5.8	0.01	−2.2	−10.6
AT3G54320	*WRI1*	AP2	0.00	−2.7	0.00	−2.8	0.00	−2.1	−7.6
AT3G44750	*HDT1*	C2H2	0.00	−3.0	0.00	−2.6	0.00	−2.0	−7.6
AT1G78600	*BBX22*	DBB	0.00	−2.9	0.00	−2.6	0.00	−2.0	−7.5
AT5G43250	*NF-YC13*	NF-YC	0.00	−2.1	0.00	−2.7	0.01	−2.1	−6.9
AT4G01580	*AT4G01580*	B3	0.00	−∞^f^	0.00	−19.0	0.00	−6.5	
AT3G57920	*SPL15*	SBP	0.00	−∞	0.00	−8.9	0.05	−2.0	
AT4G11070	*WRKY41*	WRKY	0.00	−∞	0.00	−6.4	0.04	−2.0	
AT5G13790	*AGL15*	MIKC_MADS	0.00	−3.0	0.00	−2.4	0.01	−∞	
AT5G53210	*SPCH*	bHLH	0.00	−∞	0.00	−∞	0.00	−6.9	
AT3G06120	*MUTE*	bHLH	0.00	−∞	0.00	−∞	0.00	−∞	
AT4G04450	*WRKY42*	WRKY	0.00	−∞	0.00	−∞	0.00	−∞	
AT5G43290	*WRKY49*	WRKY	0.00	−∞	0.00	−∞	0.01	−∞	

a, b *Gene locus and symbols were retrieved from TAIR10*.

c *Transcription factors were classified into families based on the assignment rules in PlantTFDB*.

d *P-value adjusted by FDR, calculated by Empirical analysis of DGE in CLC Genomics Workbench*.

e *FC—fold-change in expression of genes between data sets (comparing samples with abundant vs. no wall ingrowths), calculated by Empirical analysis of DGE in CLC Genomics Workbench*.

f *−∞ indicates gene was not expressed (exon counts <10) in sample with no detectable wall ingrowths, but was expressed (exon counts ≥10) in the comparison sample with abundant wall ingrowths*.

g *Aggregate fold-change calculated for ranking purposes. Genes with −∞ fold change were not included in this calculation and listed at the bottom of the table*.

A feature of Table 2 is the presence of five *NAC* genes among the 17 transcription factors identified as up-regulated across all three experimental comparisons. Three of the top four genes are *NACs*, including the paralogs *NAC056* and *NAC018*, which, along with *NAC029*, are all members of the Class III-2 clade (Arabidopsis clades defined as per Jensen et al., 2010, and see Supplemental Figure 3). Additionally, another 10 *NAC* genes were also listed in the 51 transcription factors showing up-regulation in two of the three experimental comparisons (Supplementary Table 2), including *NAC032, NAC019*, and *NAC072*, each of which belongs to Class III-3 of this family in Arabidopsis. Interestingly, six of the 12 *NAC* genes making up Class III-2 and III-3 were represented as up-regulated in at least two of the three experimental comparisons of wall ingrowth deposition in PP TCs (see Supplementary Figure 3). *NAC* genes associated with secondary wall formation (see Zhong and Ye, 2015) all cluster into Clade II-1 of the *NAC* family (Supplementary Figure 3), thus, a similar clustering of *NAC* genes putatively associated with wall ingrowth formation supports a role for these genes in this process.

Other classes of transcription factors were also prominent in the list of 17 shown to be up-regulated across all three comparisons. For example, the bZIP factor *AtbZIP3* showed the second highest aggregate FC score, and two MYB-related genes, *RL6* and *RL5*, also rank highly on Table 2. In total, 12 classes of Arabidopsis transcription factors made up the 17 being up-regulated across all three experimental comparisons analyzing wall ingrowth deposition in PP TCs (Table 2).

### Phenotypic analysis of selected mutants of clade III NAC transcription factors as putative regulators of wall ingrowth deposition in PP TCs

Given the strong representation of Clade III NAC-domain transcription factors in the list of 17 showing up-regulation across all three experimental comparisons of wall ingrowth deposition in PP TCs, phenotypic analysis of selected T-DNA insertional mutants for some of these genes was investigated. For this purpose, seed of the *nac056/018* double mutant and the *nac055/019/072* triple mutant were obtained, as both mutants have previously been characterized to be homozygous knockouts for each gene (*nac056/018*—Kunieda et al., 2008; *nac055/019/072*—Zheng et al., 2012). Seed of the *nac002/032* double mutant was obtained from the GABI-DUPLO collection (Bolle et al., 2013) and independently verified to be homozygous for the T-DNA insertion in each gene (see Supplementary Figure 4A). However, while RT-PCR analysis revealed that the T-DNA insertion in *NAC002* caused knockout of gene expression, the insertion in *NAC032* caused an ~60% knockdown in expression of the *NAC032* transcript (Supplementary Figure 4B). All three mutant lines were germinated on soil alongside Col-0 as wild-type and growth phenotypes were monitored, with representative images of seedlings at day 10, 17, and 25 shown in Supplementary Figure 5. Growth of the *nac002/032* double mutant approximated that of Col-0, albeit with slightly reduced rosette size at maturity (day 25), whereas *nac019/055/072* and *nac056/018* showed variations in organ size, leaf number and rosette development. Cotyledons and first leaves were smaller in *nac055/019/072* and *nac056/018*, and overall growth of the entire rosette for these two mutants was clearly reduced compared to Col-0 (Supplementary Figure 5 and see Supplementary Figure 6). Interestingly, rosettes of the *nac55/019/072* triple mutant were clearly smaller in size, containing slightly fewer leaves (approx. 10), compared to the larger rosette of *nac056/018*, with ~12 leaves by day 25 (compare Supplementary Figures 5K–L). However, the size of juvenile Leaf 1 and Leaf 2 (asterisks) for these two mutants at maturity was similar.

To quantify temporal development of these *nac* mutants, especially the time course for expansion of cotyledons and first-pair leaves, images were imported into *ImageJ* and surface area of each individual cotyledon and first leaves was measured, with results of this analysis shown in Supplementary Figure 6. In every developmental stage examined, the size of cotyledons (Supplementary Figure 6A) and first-pair leaves (Supplementary Figure 6B) in *nac055/019/072* (gray bars) and *nac056/018* (yellow bars) were significantly smaller than in Col-0 (blue bars) (*P* < 0.01 or *P* < 0.001, student's *t*-test, *n* = 3–5), while no differences were identified for cotyledon or leaf size between *nac002/032* (orange bars) and Col-0. For Col-0 and all mutant lines, cotyledons and leaves dramatically expanded during early stages of development, but without significant difference between days 14 and 17 for cotyledons (Supplementary Figure 6A) or between days 21 and 25 for first leaves (Supplementary Figure 6B), suggesting that cotyledons and first leaves for all lines were fully expanded by days 17 and 25, respectively. Also, cotyledon size in *nac055/019/072* and *nac056/018* plants reached only half that compared to Col-0, whereas first leaves of these two mutants achieved about two-thirds the size of Col-0 and the *nac002/032* mutant (Supplementary Figure 6).

**Figure 7:**
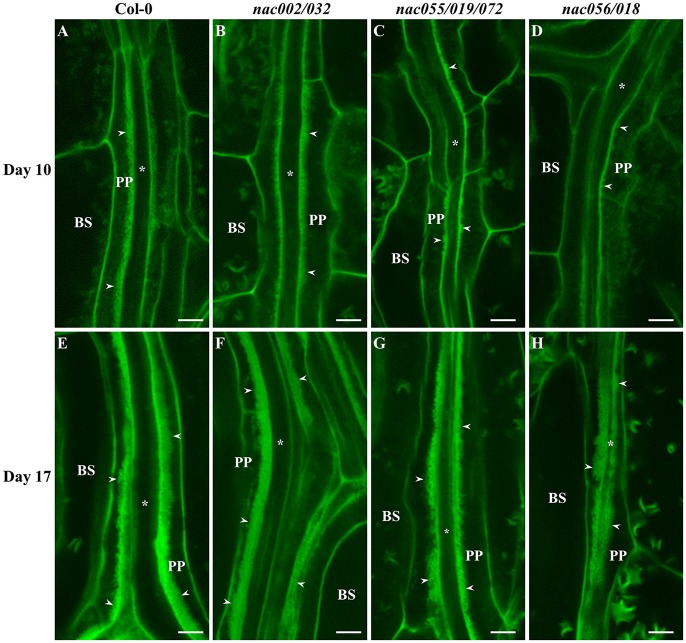
Confocal imaging of wall ingrowth deposition in PP TCs of Arabidopsis cotyledons from Col-0 and *nac* mutants. By day 10, PP TCs in cotyledons from both Col-0 **(A)** and *nac002/032* **(B)** showed extensive wall ingrowth deposition along the face of PP TCs adjacent to SE/CCs. In contrast, evidence of wall ingrowth deposition was detected only as discrete patches in PP TCs from cotyledons of *nac055/019/072* **(C)** or as small projections in *nac056/018* **(D)**. At day 17, wall ingrowths in PP TCs of cotyledons from Col-0 **(E)**, *nac002/032* **(F)**, *nac055/019/072* **(G)** and *nac056/018* **(H)** were highly abundant (Class V; 7–8 points) and occupied a considerable volume of the PP TC. BS denotes bundle sheath cells; asterisks indicate SE/CCs; PP denotes PP cell or PP TC, as appropriate; arrowheads indicate wall ingrowths in PP TCs. The crescent-shaped fluorescent fragments occasionally seen in BS cells correspond to remnant starch grains not completely extracted by the bleach treatment. Scale bars = 5 μm.

**Figure 8:**
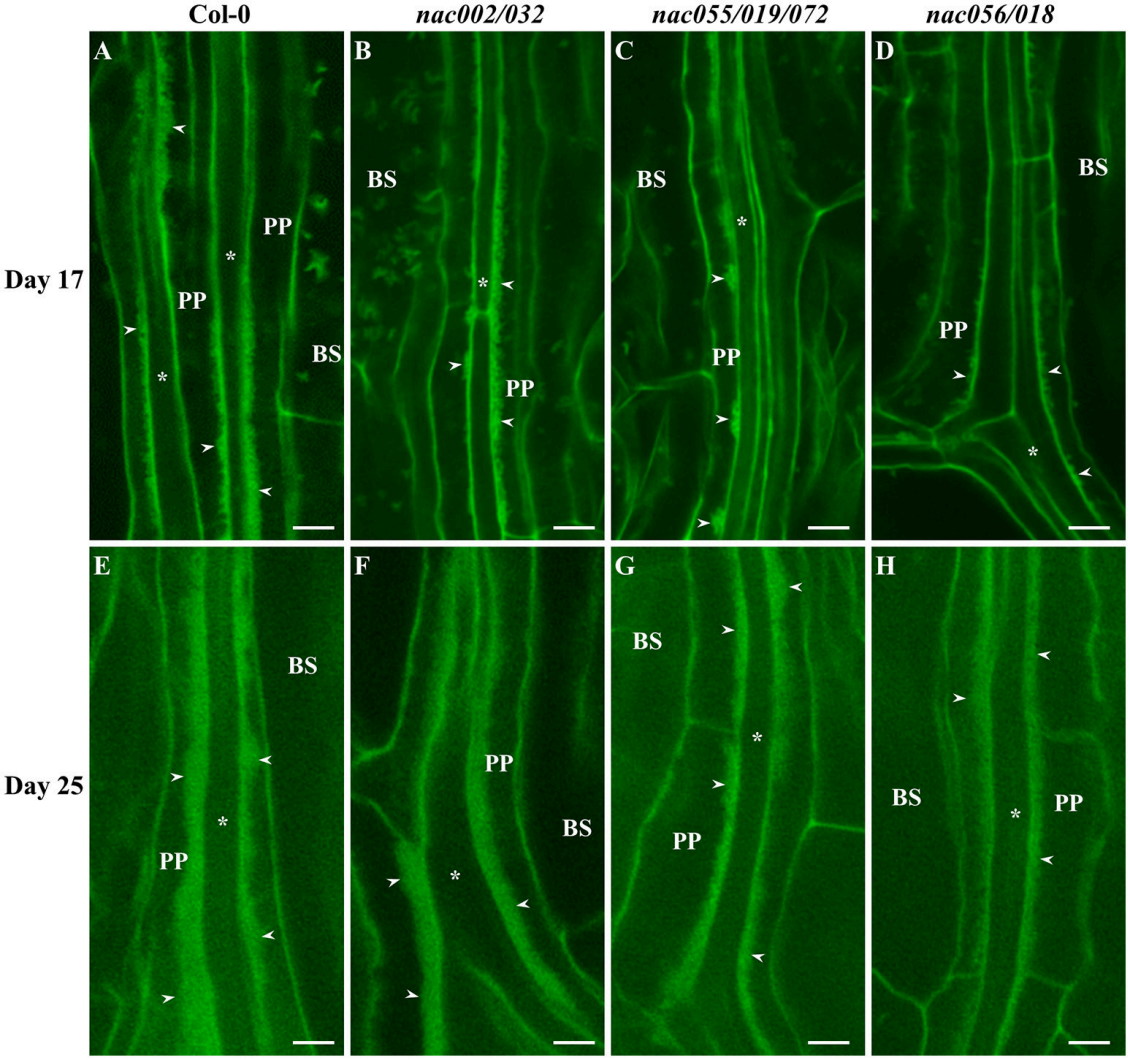
Confocal imaging of wall ingrowth deposition in PP TCs of Arabidopsis Leaf 1 and 2 from Col-0 and *nac* mutants. By day 17, continuous bands of wall ingrowth deposition were seen in PP TCs from first leaves of both Col-0 **(A)** and *nac002/032* **(B)**. In contrast, wall ingrowths in *nac055/019/072* were deposited as mostly discontinuous patches or clumps **(C)**, or as discrete projections in *nac056/018* **(D)**. By day 25, wall ingrowths in Col-0 **(E)** and *nac002/032* **(F)** were seen as thick bands representing massive deposition (Class V; 7–8 points), while bands in *nac055/019/072* **(G)** and *nac056/018* **(H)** appeared to be thinner and occupied less volume of the PP TCs (Class IV; 5–6 points). BS denotes bundle sheath cells; asterisks indicate SE/CCs; PP denotes PP cell or PP TC, as appropriate; arrowheads indicate wall ingrowths in PP TCs. The fluorescent fragments occasionally seen in BS cells correspond to remnant starch grains not completely extracted by the bleach treatment. Scale bars = 5 μm.

To assess wall ingrowth deposition in PP TCs of these *nac* mutants compared to Col-0 as wild-type, cotyledons and first-pair leaves were harvested from plants at days 10 and 17 for cotyledons, and days 17 and 25 for first leaves. Days 10 and 17 for cotyledons and first leaves, respectively, are time-points where wall ingrowth deposition in PP TCs is abundant, and days 17 and 25 are time-points when both cotyledons and first leaves, respectively, are fully expanded (Supplementary Figure 6). Representative confocal images of wall ingrowth deposition in mPS-PI-stained tissues from Col-0 and the *nac* mutants are shown in Figure 7 for cotyledons and Figure 8 for first-pair leaves. As seen in Figure 7, by day 10, wall ingrowth deposition was abundant in cotyledon PP TCs in both Col-0 and the *nac002/032* double mutant, this appearing as continuing linear bands of wall ingrowths along the face of the PP TC adjacent to SE/CCs (Figures 7A,B). In comparison, only discrete patches and sometimes dot-like fluorescence corresponding to wall ingrowth deposition was occasionally observed in PP TCs of cotyledons from the *nac055/019/072* triple mutant and the *nac056/018* double mutant, respectively (Figures 7C,D). By day 17, however, massive deposition of wall ingrowths seen as thick bands occupying a considerable volume of each PP TCs was seen in Col-0 (Figure 7E) and *nac002/032* (Figure 7F), and apparently similar levels in *nac055/019/072* (Figure 7G) and *nac056/018* (Figure 7H).

**Figure 9:**
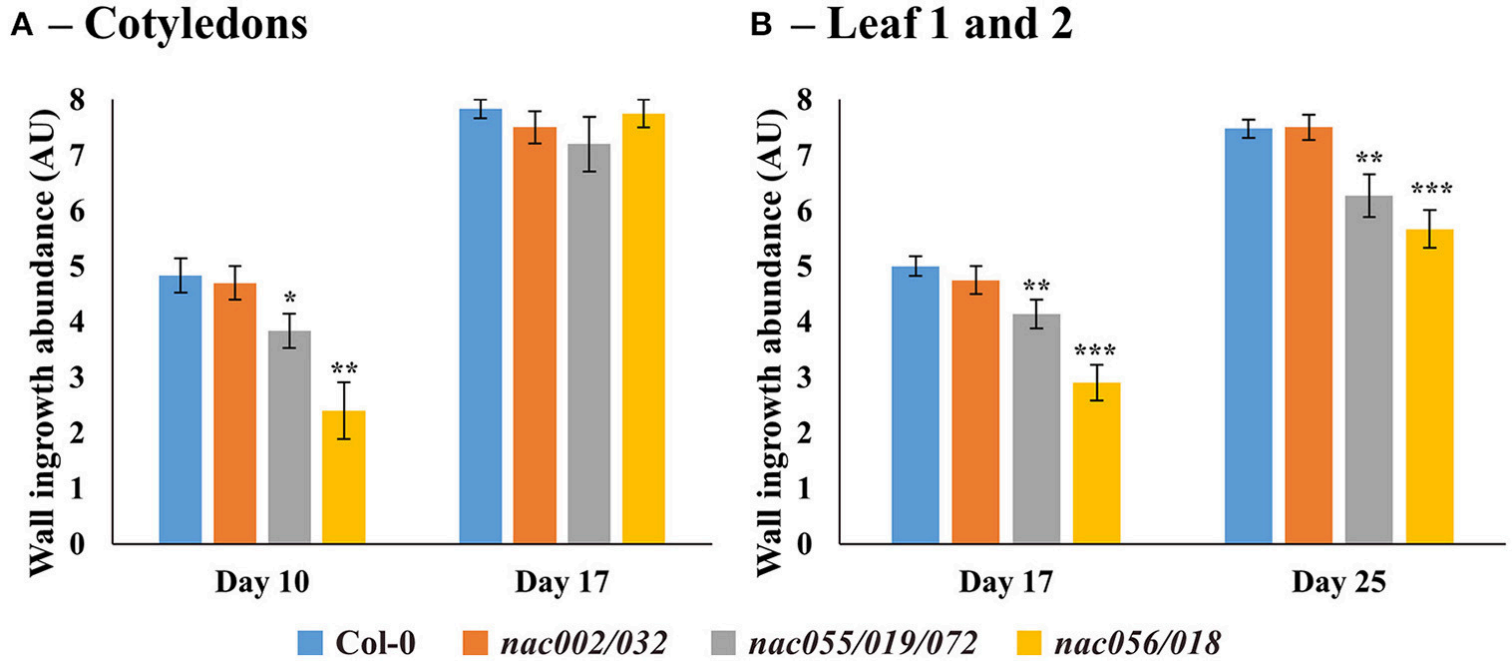
Semi-quantitative analysis of wall ingrowth deposition in Arabidopsis cotyledons and first leaves from Col-0 and *nac* mutants at selected developmental stages. **(A)** In cotyledons, by day 10 wall ingrowth deposition in both Col-0 and *nac002/032* was indistinguishable, whereas deposition was significantly decreased in both *nac055/019/072* (*P* < 0.05) and *nac056/018* (*P* < 0.01). By day 17, however, no difference in wall ingrowth abundance was observed in any *nac* mutant compared to Col-0. **(B)** For Leaf 1 and 2 at day 17, wall ingrowth deposition in both Col-0 and *nac002/032* was indistinguishable, whereas deposition was significantly decreased in both *nac055/019/072* (*P* < 0.01) and *nac056/018* (*P* < 0.001). By day 25, however, again, no differences in wall ingrowth abundance were seen in *nac002/032* compared to Col-0, but abundance was significantly reduced in *nac055/019/072* (*P* < 0.01) and *nac056/018* (*P* < 0.001) compared to Col-0. Data shows mean ± SE of scores for wall ingrowth deposition in arbitrary units according to the classification scheme of Nguyen et al. (2017). ^*^P < 0.05; ^**^P < 0.01; ^***^P < 0.001, student's *t*-test comparing wall ingrowth scores in each mutant line compared to Col-0, at each developmental stage; *n* = 5–10.

Similar to deposition in cotyledons, wall ingrowths in PP TCs of first-pair leaves were abundant by day 17 in both wild-type and *nac002/032* (Figures 8A,B), whereas in *nac055/019/072* deposition was generally reduced and localized to discrete clumps (Figure 8C), or more limited in *nac056/018* compared to Col-0 and often seen as smaller, finger-like projections (Figure 8D). At day 25, both Col-0 and *nac002/032* displayed massive deposition of wall ingrowths (Figures 8E,F), while such bands appeared to be thinner and clearly less abundant relative to cell size in *nac055/019/072* and *nac056/018*, although they were still deposited in a continuous manner (Figures 8G,H). Semi-quantitative scoring of wall ingrowth deposition in PP TCs of the three *nac* mutants compared to Col-0 was performed as described in Methods (and see Nguyen et al., 2017). As shown in Figure 9, and consistent with the qualitative observations, wall ingrowth deposition in the *nac002/032* double mutant (orange bars) scored similarly to Col-0 (blue bars) in cotyledons of day 10 plants (Figure 9A). In contrast, significant reductions in wall ingrowth abundance were seen in the *nac055/019/072* triple mutant (gray bars; *P* < 0.05) and particularly in the *nac056/018* double mutant (yellow bars; *P* < 0.01) compared to Col-0 at this early stage of PP TC development (Figure 9A). In contrast to these differences at day 10, by day 17 in cotyledons, no differences were seen for wall ingrowth deposition in any of the three *nac* mutants compared to Col-0 (Figure 9A). In immature first leaves (day 17), a similar conclusion to that seen for immature cotyledons was recorded whereby the double mutant *nac002/032* was indistinguishable from Col-0, but significant decreases were observed in *nac055/019/072* (*P* < 0.01) and especially in *nac056/018* (*P* < 0.001) (Figure 9B). At day 25, however, and different from mature cotyledons, the differences seen in wall ingrowth deposition in both *nac055/019/072* and *nac056/018* remained statistically significant (*P* < 0.01; *P* < 0.001, respectively) compared to Col-0 (Figure 9B). Collectively, these results support the conclusion that *NAC056* and its paralog *NAC018*, and possibly to a lesser extent *NAC055, NAC019*, and *NAC072*, may be involved in regulating wall ingrowth deposition in PP TCs, and that this regulation may operate redundantly and possibly differentially between cotyledons and juvenile leaves.

**Figure 10:**
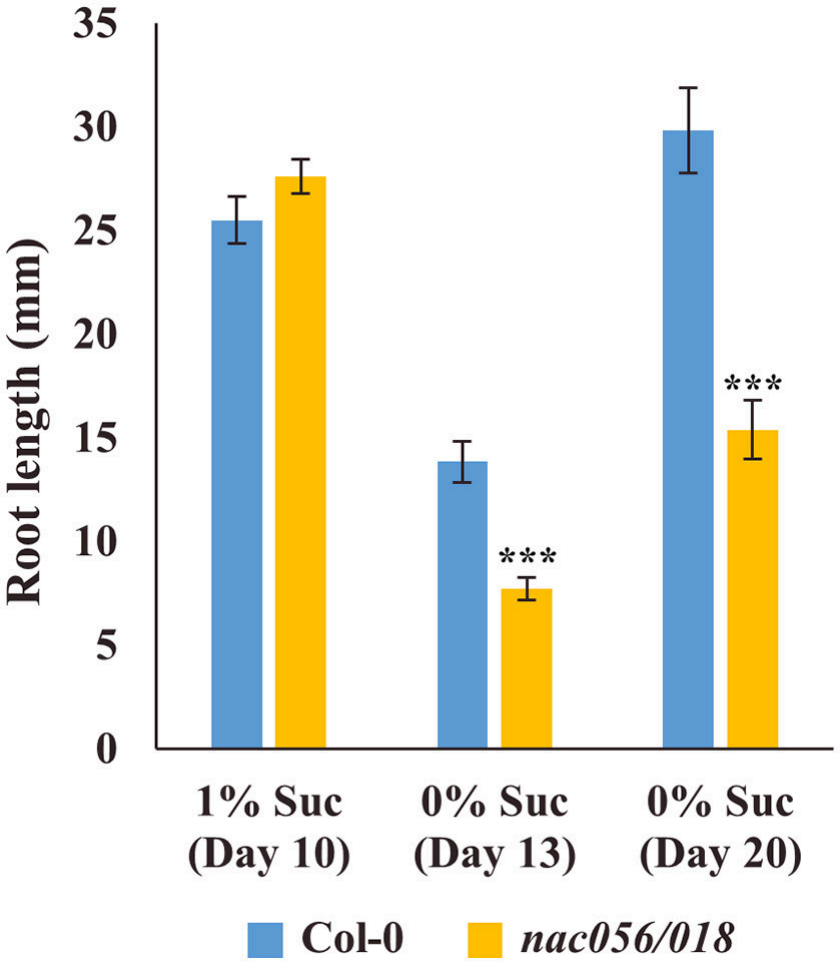
Root growth assay of *nac056/018* mutant in presence or absence of added sucrose. Seeds of Col-0 or *nac056/018* were grown on nutrient agar plates in the presence or absence of 1% (w/v) sucrose. After 10 days in the presence of sucrose, root growth in the two lines was comparable. At 13 days in the absence of sucrose, root growth of *nac056/018* was significantly reduced compared to Col-0, and this reduction continued for roots grown for 20 days in the absence of sucrose. Data shows mean ± SE for root length, student's *t*-test, ^***^P < 0.001, *n* ≥ 15.

To test the hypothesis that reduced levels of wall ingrowth deposition in PP TCs may impact phloem loading and consequent sugar transport to roots, root growth of Col-0 and *nac056/018* was measured in the presence or absence of sucrose. At 10 days in the presence of 1% (w/v) sucrose, root growth of *nac056/018* was comparable to Col-0, however by 13 and 20 days in the absence of sucrose, *nac056/018* root growth was significantly reduced compared to Col-0 (*P* < 0.001, student's *t-*test, *n* ≥ 15) (Figure 10). This result supports the conclusion that reduced wall ingrowth development in PP TCs of Arabidopsis cotyledons of the *nac056/018* double mutant negatively impacted phloem loading capacity in these plants.

## Discussion

### Temporal and spatial analysis of PP TC development

In this study we identified and semi-quantified distinct temporal and spatial profiles of wall ingrowth deposition in PP TCs of Arabidopsis cotyledons, juvenile leaves and adult leaves, and used these profiles for RNA-Seq to identify potential regulators of PP TC development. Prominent amongst the list of transcription factors identified as commonly up-regulated across these profiles were Clade III members of the NAC-domain family, in particular the Clade III-2 paralogs *NAC056* and *NAC018*, and the closely related *NAC029*. Phenotypic analysis of the *nac056/018* double mutant, and *nac055/019/072*, a triple mutant of Clade III-3 *NAC* genes up-regulated across TC development, revealed significant reductions in levels of wall ingrowth deposition in PP TCs of cotyledons and juvenile leaves, suggesting that these genes may have important roles in regulating deposition of wall ingrowths in PP TCs.

A key enabling strategy in this study was using confocal imaging of mPS-PI-stained tissues to semi-quantitatively map wall ingrowth deposition in PP TCs across developing cotyledons and leaves. Nguyen et al. (2017) developed these procedures to compare wall ingrowth deposition in leaf veins across shoot development, noting the extent of deposition varied substantially between juvenile and adult leaves, leading to the conclusion that wall ingrowth deposition in PP TCs represents a novel trait of heteroblasty. The current study extended these observations by analyzing wall ingrowth deposition across developing cotyledons and juvenile leaves, as well as across the basipetal gradient of ingrowth deposition in minor veins of mature adult leaves. The observed basipetal gradient of wall ingrowth deposition seen in adult leaves is consistent with the sink/source transition in a developing leaf (Robinson-Beers et al., 1990; Imlau et al., 1999), thus implicating PP TCs in phloem loading in mature Arabidopsis leaves (Haritatos et al., 2000; Chen et al., 2012). Interestingly, some degree of basipetal development of PP TCs was observed in developing cotyledons and juvenile Leaf 1 and 2, but the gradient was quickly lost as these organs developed (Figure 3). Collectively, therefore, combining transcript profiling (RNA-Seq) across three different examples of wall ingrowth deposition (i.e., cotyledons, juvenile leaves, adult leaves) provided an opportunity to identify key factors involved in regulating this process. While harvesting whole organs for this analysis potentially limits the ability to identify transcriptional changes presumably occurring specifically in PP TCs, this limitation is off-set by comparing wall ingrowth deposition across the three different organ types. Cotyledons produced during embryogenesis are considered to be a particular leaf type that arise from a part of the shoot apical meristem that also gives rise to rosette leaves (Aida et al., 1997; Conway and Poethig, 1997; Kaplan and Cooke, 1997; Tsukaya et al., 2000). However, genetic analyses indicate that cotyledons do not share all genetic programs involved in the development of leaves (Van Lijsebettens and Clarke, 1998; Tsukaya et al., 2000; Chandler, 2008), as exampled by simpler vascular patterning compared to leaves (Sieburth and Deyholos, 2006). Furthermore, heteroblastic leaf forms, i.e., juvenile vs. adult leaves, become distinct very early in development and display qualitative differences in patterns of cellular differentiation, leading to the conclusion that juvenile and adult leaves are specified by different developmental programs (Kerstetter and Poethig, 1998). Furthermore, Nguyen et al. (2017) established that the differentiation of xylem and SE/CC complex in juvenile leaves and cotyledons occurs prior to the *trans-*differentiation of PP TCs, thus gene expression events associated with the latter may not overlap greatly with those of the former. Collectively, therefore, at least some of the differential gene expression events occurring commonly across the three experimental profiles of TC development analyzed here may be expected to correspond to genes regulating wall ingrowth deposition in PP TCs.

### RNA-seq analysis identified transcription factors commonly up-regulated across temporal and spatial deposition of wall ingrowths in PP TCs

A total of 68 transcription factors were identified as commonly up-regulated in at least two of the three RNA-Seq experimental comparisons, and of this total, 17 transcription factors were commonly up-regulated in all three comparisons (Table 2). A feature of this list was the high representation of Class III-2 *NAC* genes (*NAC056, NAC018, NAC029*), each of which ranked in the top four based on aggregate fold-change across the three experimental comparisons. In addition to these NACs, another three (*NAC072, NAC032, NAC019*) of the ten appearing in the list of transcription factors up-regulated across two out of the three experimental comparisons, belong to the Class III-3 subclade (Supplementary Table 2 and Supplementary Figure 3). Interestingly, this representation of *NACs* amongst the cohort of commonly up-regulated genes is 2.4-fold higher than that expected based on the size of the *NAC* family compared to all other transcription factors in Arabidopsis. This observation, along with the earlier analysis that 50% of the Class III NAC transcription factors were up-regulated across wall ingrowth deposition, supports a regulatory role for this class of genes in PP TC development. This phylogenetic association of *NAC* genes with wall ingrowth deposition is similar in some degree to that seen for *VNS* genes involved in regulating secondary wall deposition (see Endo et al., 2015; Zhong and Ye, 2015), which all cluster in Clade II-1 of the NAC family (Supplementary Figure 3). This observation reveals strongly conserved structure/function relationships in regulating secondary wall biosynthesis by these *VNS* genes and suggests an evolutionally common ancestral capacity in regulating cell wall modification during differentiation of specific cell types (Nakano et al., 2015). Therefore, the substantial phylogenetic clustering of *NAC* genes identified as commonly up-regulated across wall ingrowth deposition in PP TCs also suggests co-involvement in a common developmental function, in this case putatively regulating wall ingrowth deposition in PP TCs.

### Phenotypic analysis showed reduced wall ingrowth deposition and compromised root growth in *NAC* mutants

A role for the *NAC* genes listed in Table 2 and Supplementary Table 2 in regulating wall ingrowth deposition in PP TCs was tested using relevant T-DNA insertional mutants. Analysis of two double mutants (*nac056/018* and *nac032/002*) and one triple mutant (*nac055/019/072*) revealed that while wall ingrowth abundance in PP TCs of the double mutant *nac002/032* was similar to Col-0 in both cotyledons and leaves, significant reductions were seen in the other two mutants, particularly *nac056/018*, which showed age- and tissue-dependent differences compared to wild-type. For example, in early stages of both cotyledon and juvenile leaf development, *nac056/018* showed an ~50% reduction in wall ingrowth deposition compared to Col-0. By maturity in these organs, however, wall ingrowth deposition remained significantly reduced in juvenile leaves but not different in cotyledons (Figure 9). A smaller reduction was seen in *nac055/019/072*, which again persisted in leaves but not mature cotyledons. The reductions in wall ingrowth development seen in both *nac056/018* and *nac055/019/072* might simply be a consequence of reduced organ size compared to wild-type (Supplementary Figure 6). However, in the recent study by Nguyen et al. (2017) investigating PP TC development as a novel trait of heteroblasty, no correlations were observed between cotyledon/leaf size and wall ingrowth abundance. Therefore, the results reported here support the conclusion that significant reductions seen in wall ingrowth deposition in the *nac056/018* and *nac055/019/072* mutants is related to loss of gene function regulating this process rather than an indirect effect caused by reduced organ growth. Conclusions regarding a role for either *NAC002* or *NAC032* in regulating wall ingrowth deposition were inconclusive since the *nac002/032* double mutant was not a complete knock-out for *NAC032* (Supplementary Figure 4), and thus gene redundancy may have masked any effects on ingrowth deposition.

In Arabidopsis, wall ingrowth deposition in PP TCs is proposed to facilitate phloem loading of source leaves (Haritatos et al., 2000; Maeda et al., 2006). Root growth was used as a measure of phloem export capacity to test the effect of knocking out both *ATSWEET11* and *12* in PP TCs (Chen et al., 2012), and similar to their study, the *nac056/018* double mutant also showed a sucrose-dependent reduction in root growth (Figure 10). This result is consistent with the proposal that wall ingrowths in PP TCs increases phloem loading capacity in developing cotyledons and juvenile leaves. In this context, Kircher and Schopfer (2012) demonstrated that developing cotyledons act as the source of photoassimilates to supply seedling growth, in particular root growth. The reductions in wall ingrowth deposition seen in early cotyledon development (Figure 9A) and the effects seen on sucrose-dependent root growth in *nac056/018* (Figure 10), is consistent with wall ingrowths playing this role, particularly in early stages of cotyledon and juvenile leaf development.

*NAC018* was one of the seven novel *NAC* genes identified through co-expression network analysis of cell wall-related genes in Arabidopsis (Wang et al., 2012), and indeed, *NAC018* (also named *NAC-REGULATED SEED MORPHOLOGY2—NARS2*) and its paralog *NAC056* (*NARS1*), redundantly regulate seed morphology and embryogenesis, as well as differentiation of seed coat mucilage (Kunieda et al., 2008; Tsai et al., 2017). While a large amount of mucilage was accumulated around the columella in epidermal cells of wild-type seed coats, the double mutant *nac056/018* (*nars1/nars2*) failed to form columella or mucilage (Kunieda et al., 2008). Mucilage contains a special type of secondary cell wall that is enriched in pectins (Kunieda et al., 2008; Arsovski et al., 2010; Haughn and Western, 2012; Voiniciuc et al., 2015), which is also a predominant component in wall ingrowths in TCs (Gunning and Pate, 1974; Vaughn et al., 2007). Collectively, these observations support the hypothesis that *NAC056*/*NAC018* could also be involved in TC biology, possibly by regulating the pectin component of wall ingrowths.

The triple mutant *nac019/055/072* was used to test the involvement of the Clade III-3 *NAC019* and *NAC072* genes, both of which appeared on the list of genes up-regulated in two of the three experimental comparisons examining wall ingrowth deposition in PP TCs of Arabidopsis (Supplementary Table 2). All three members of this group are well characterized in a broad range of processes including responses to drought and salinity stress, abscisic acid, ethylene and methyl jasmonate signaling, as well as pathogen defense and senescence (Fujita et al., 2004; Tran et al., 2004; Bu et al., 2008; Jensen et al., 2010; Zheng et al., 2012; Hickman et al., 2013; Schweizer et al., 2013). Many of these abiotic and biotic stress responses are known to influence TC development (see Andriunas et al., 2013), and thus the three *NAC* genes may be components of overlapping genetic networks regulating such events. In this context, the significant reductions in wall ingrowth abundance seen in developing cotyledons and in both developing and mature juvenile leaves (Figure 9) support this conclusion. In a similar manner, the *nac002/032* double mutant was examined since *NAC032* was up-regulated in two of the three experimental comparisons undertaken (Supplementary Table 2), and a link to TC biology for *NAC002* is suggested by the observation that its *V. faba* ortholog, *VfNAC002*, was identified as a candidate transcriptional regulator of epidermal TC development in *V. faba* cotyledons (Arun-Chinnappa and McCurdy, 2016). Both *NAC*s are also involved in abiotic and biotic stress responses (Wu et al., 2009; Vermeirssen et al., 2014; Allu et al., 2016; Mahmood et al., 2016). However, as discussed above, the double mutant was only a partial knock-down for *NAC032*, thus gene redundancy may have masked a wall ingrowth phenotype in *nac002/032*.

### Roles for other transcription factors in regulating wall ingrowth deposition

Other genes of interest emerging from this analysis include *AtbZIP3*, which showed the second highest aggregate fold-change score of genes up-regulated across all three experimental comparisons of wall ingrowth deposition (Table 2). This little-known S-group member of the large bZIP family in Arabidopsis is down-regulated by glucose (Matiolli et al., 2011), a sugar proposed to suppress wall ingrowth deposition in TCs (see Andriunas et al., 2013). *AtbZIP3* was also identified as a cell wall related gene in Arabidopsis based on co-expression network analysis (Wang et al., 2012), and another *AtbZIP* gene, *GBF3* (Supplementary Table 2), was also identified in a protein-DNA network generated between Arabidopsis transcription factors and secondary wall metabolic genes (Taylor-Teeples et al., 2015). A third member of the family, *TGA7* (Supplementary Table 2), which shows high expression in vascular bundles compared to mesophyll (Endo et al., 2014), was reported to be differentially up-regulated under cold treatment in the tocopherol mutant *vte2*, which responds to cold by increased deposition of wall ingrowths in PP TCs (Maeda et al., 2014).

In addition to the prominence of bZIP genes, a finding emerging from this study was the relatively high number of genes previously associated with cell wall biosynthesis appearing as up-regulated across wall ingrowth deposition in PP TCs. For example, four *MYB* genes, namely *MYB59* (Table 2), *AT3G11280, MYB28*, and *MYB29* (Supplementary Table 2) have all been identified as components of a gene network regulating secondary wall biosynthesis (Cassan-Wang et al., 2013; Taylor-Teeples et al., 2015). Similarly, *NAC018* (Table 2), *NAC048, NAC092*, and *WRKY26* (Supplementary Table 2) have also been identified as cell wall-related genes (Wang et al., 2012; Taylor-Teeples et al., 2015), while *NAC056, NAC032*, and *NAC048* are induced in giant cells (identified from public microarray dataset GSE37553; https://www.ncbi.nlm.nih.gov/geo/query/acc.cgi?acc=GSE37553) and *NAC019* is up-regulated during syncytia formation (Szakasits et al., 2009), both of which display TC-like characteristics (Cabrera et al., 2014; Rodiuc et al., 2014).

In addition to the predominantly *MYB* and *NAC* genes described above, members from other families listed in Table 2 and Supplementary Table 2 are also identified to be cell wall- or secondary wall-related, such as the ERF members *RAP2-3* (Table 2), *ERF003, ERF060*, and *RAP2-10* (Supplementary Table 2) (Wang et al., 2012; Cassan-Wang et al., 2013; Taylor-Teeples et al., 2015). *RAP2-3* is also up-regulated in syncytia (Szakasits et al., 2009). Interestingly, *bHLH62* (Supplementary Table 2) which was identified as a cell wall related gene in Arabidopsis based on co-expression network analysis (Wang et al., 2012), has an ortholog in *V. faba* displaying epidermal-specific up-regulation in epidermal TCs of cultured *V. faba* cotyledons (Arun-Chinnappa and McCurdy, 2016). Another *bHLH* gene, *AT1G15790* (Supplementary Table 2), was induced in both syncytia and giant cells (Szakasits et al., 2009; Barcala et al., 2010).

Collectively, the RNA-Seq analysis described here has identified numerous genes associated with cell wall biology in the cohort of genes up-regulated across wall ingrowth deposition in PP TCs. This observation is consistent with the conclusion that some aspects of wall ingrowth deposition may be similar to secondary wall formation, whereby a hierarchical cascade of gene expression is responsible for directing the cellulose/hemicellulose component of secondary walls distinct from that required for lignin biosynthesis. Thus commonalities between the cellulose/hemicellulose pathways for both wall ingrowth and secondary wall deposition may exist.

### Down-regulated genes: an additional contribution to transcriptional regulation of PP TC development?

In this study differentially expressed genes were identified as both up- and down-regulated. While the conceptual focus of this research has been to identify transcription factors actively “switching on” the genetic network required for wall ingrowth deposition, a role for negative regulators, or suppressors, of TC development is entirely feasible. Again, using secondary wall biosynthesis as a model, genes such as *NAC083* and *NAC104* function as negative regulators of secondary wall biosynthesis in Arabidopsis xylem vessel formation (Zhao et al., 2008; Yamaguchi et al., 2010), while several MYB transcription factors and their orthologous genes are reported to negatively regulate lignin biosynthesis and wood generation in Arabidopsis and other species such as switchgrass, maize and Eucalyptus (Sonbol et al., 2009; Fornalé et al., 2010; Legay et al., 2010; Shen et al., 2012).

The list of genes commonly down-regulated across PP TC development represents a more diverse collection of families, albeit with four of the 23 in total belonging to the bHLH family (Table 3). *bHLH* genes have important roles in regulating cell proliferation to cell lineage establishment (Toledo-Ortiz et al., 2003); for example, *bHLH12*, showing the highest aggregate negative fold-change score as listed in Table 3, controls trichrome cell fate determination (Symonds et al., 2011), while *SPCH* and *MUTE* (Table 3), regulate stomatal development (Simmons and Bergmann, 2016). The differentiation fate for these cell types is determined earlier than the time points selected here to study wall ingrowth deposition, thus their identification as down-regulated genes in this analysis could be expected and thus any relevance to regulating TC development may be minimal. Similarly, most of the secondary wall master regulators (Supplementary Table 3; see Zhong and Ye, 2015) were commonly down-regulated in this study, consistent with the observation that xylem and phloem differentiation is complete prior to the *trans*-differentiation of PP cells into PP TCs (Nguyen et al., 2017). In contrast, however, the recent paper identifying development of PP TCs as a novel trait of heteroblasty also reported that levels of wall ingrowth deposition were positively correlated with the miR156-mediated repression of *SQUAMOSA PROMOTER BINDING PROTEIN LIKE* (*SPL*) genes (Nguyen et al., 2017). Among this small family of transcription factors, *SPL9, 10*, and *15* (i.e., *SPL9-*Group *SPLs*) displayed the strongest negative correlation with PP TC development, and relevant over-expression lines exhibited lower levels of wall ingrowth deposition compared to wild-type (Nguyen et al., 2017). The RNA-Seq analysis reported here showed that *SPL15* is commonly down-regulated in all three experimental comparisons (Table 3), thus providing strong support for the notion that *SPL9-*group *SPL*s function as negative regulators of TC development in Arabidopsis.

## Conclusions and future directions

This study identified a cohort of Class III *NAC* genes commonly up-regulated across wall ingrowth deposition in PP TCs in three different organs, namely cotyledons, juvenile leaves and adult leaves. The disruption of wall ingrowth deposition in the double mutant *nac056/018* and to a lesser extent *nac055/019/072*, supports the conclusion that these genes may be involved in the regulatory cascade of gene expression required for wall ingrowth biosynthesis. Complementation of the wall ingrowth phenotype in these mutants by over-expression of wild-type genes under control of a PP-specific promoter will be a useful approach to further dissect the role of these *NAC* genes in regulating wall ingrowth deposition. Furthermore, several other cohorts of genes, many identified previously to be associated with cell wall synthesis in Arabidopsis, supports the conclusion that the experimental strategy used in this study was successful in revealing candidate genes putatively involved in regulating wall ingrowth deposition in PP TCs.

## Author contributions

DM: designed the experimental approach; YW, JH, FY, and SN: generated the experimental data and provided analysis; YW and DM: wrote the manuscript and all authors commented on the final draft.

### Conflict of interest statement

The authors declare that the research was conducted in the absence of any commercial or financial relationships that could be construed as a potential conflict of interest.

## References

[B1] Aida M. Ishida T. Fukaki H. Fujisawa H. Tasaka M. (1997). Genes involved in organ separation in Arabidopsis: an analysis of the cup-shaped cotyledon mutant. Plant Cell 9, 841–857. 10.1105/tpc.9.6.841 9212461 PMC156962

[B2] Allu A. D. Brotman Y. Xue G. P. Balazadeh S. (2016). Transcription factor ANAC032 modulates JA/SA signalling in response to *Pseudomonas syringae* infection. EMBO Rep. 17, 1578–1589. 10.15252/embr.201642197 27632992 PMC5090710

[B3] Amiard V. Demmig-Adams B. Mueh K. E. Turgeon R. Combs A. F. Adams W. W. III. (2007). Role of light and jasmonic acid signaling in regulating foliar phloem cell wall ingrowth development. New Phytol. 173, 722–731. 10.1111/j.1469-8137.2006.01954.x 17286821

[B4] Amiard V. Mueh K. E. Demmig-Adams B. Ebbert V. Turgeon R. Adams W. W. III. (2005). Anatomical and photosynthetic acclimation to the light environment in species with differing mechanisms of phloem loading. Proc. Natl. Acad. Sci. U.S.A. 102, 12968–12973. 10.1073/pnas.0503784102 16120679 PMC1200274

[B5] Andriunas F. A. Zhang H. M. Weber H. McCurdy D. W. Offler C. E. Patrick J. W. (2011). Glucose and ethylene signalling pathways converge to regulate *trans*-differentiation of epidermal transfer cells in *Vicia narbonensis* cotyledons. Plant J. 68, 987–998. 10.1111/j.1365-313X.2011.04749.x 21848654

[B6] Andriunas F. A. Zhang H. M. Xia X. Offler C. E. McCurdy D. W. Patrick J. W. (2012). Reactive oxygen species form part of a regulatory pathway initiating trans-differentiation of epidermal transfer cells in *Vicia faba* cotyledons. J. Exp. Bot. 63, 3617–3629. 10.1093/jxb/ers029 22442421 PMC3388844

[B7] Andriunas F. A. Zhang H. M. Xia X. Patrick J. W. Offler C. E. (2013). Intersection of transfer cells with phloem biology—broad evolutionary trends, function, and induction. Front. Plant Sci. 4:221. 10.3389/fpls.2013.00221 23847631 PMC3696738

[B8] Arsovski A. A. Haughn G. W. Western T. L. (2010). Seed coat mucilage cells of *Arabidopsis thaliana* as a model for cell wall research. Plant Signal. Behav. 5, 796–801. 10.4161/psb.5.7.11773 20505351 PMC3014532

[B9] Arun-Chinnappa K. S. McCurdy D. W. (2016). Identification of candidate transcriptional regulators of epidermal transfer cell development in *Vicia faba* cotyledons. Front. Plant Sci. 7:717. 10.3389/fpls.2016.00717 27252730 PMC4879131

[B10] Arun Chinnappa K. S. Nguyen S. Hou J. Wu Y. McCurdy D. W. (2013). Phloem parenchyma transfer cells in Arabidopsis – an experimental system to identify transcriptional regulators of wall ingrowth formation. Front. Plant Sci. 4:102. 10.3389/fpls.2013.00102 23630536 PMC3634129

[B11] Barcala M. García A. Cabrera J. Casson S. Lindsey K. Favery B.. (2010). Early transcriptomic events in microdissected Arabidopsis nematode-induced giant cells. Plant J. 61, 698–712. 10.1111/j.1365-313X.2009.04098.x 20003167

[B12] Bolle C. Huep G. Kleinbölting N. Haberer G. Mayer K. Leister D.. (2013). GABI-DUPLO: a collection of double mutants to overcome genetic redundancy in *Arabidopsis thaliana*. Plant J. 75, 157–171. 10.1111/tpj.12197 23573814

[B13] Bu Q. Jiang H. Li C. B. Zhai Q. Zhang J. Wu X.. (2008). Role of the *Arabidopsis thaliana* NAC transcription factors ANAC019 and ANAC055 in regulating jasmonic acid-signaled defense responses. Cell Res. 18, 756–767. 10.1038/cr.2008.53 18427573

[B14] Cabrera J. Barcala M. Fenoll C. Escobar C. (2014). Transcriptomic signatures of transfer cells in early developing nematode feeding cells of Arabidopsis focused on auxin and ethylene signaling. Front. Plant Sci. 5:107. 10.3389/fpls.2014.00107 24715895 PMC3970009

[B15] Cassan-Wang H. Goué N. Saidi M. N. Legay S. Sivadon P. Goffner D.. (2013). Identification of novel transcription factors regulating secondary wall formation in Arabidopsis. Front. Plant Sci. 4:189. 10.3389/fpls.2013.00189 23781226 PMC3677987

[B16] Chandler J. W. (2008). Cotyledon organogenesis. J. Exp. Bot. 59, 2917–2931. 10.1093/jxb/ern167 18612170

[B17] Chen L. Q. Qu X. Q. Hou B. H. Sosso D. Osorio S. Fernie A. R.. (2012). Sucrose efflux mediated by SWEET proteins as a key step for phloem transport. Science 335, 207–211. 10.1126/science.1213351 22157085

[B18] Conway L. J. Poethig R. S. (1997). Mutations of *Arabidopsis thaliana* that transform leaves into cotyledons. Proc. Natl. Acad. Sci. U.S.A. 94, 10209–10214. 10.1073/pnas.94.19.10209 9294189 PMC23341

[B19] Dibley S. J. Zhou Y. Andriunas F. A. Talbot M. J. Offler C. E. Patrick J. W.. (2009). Early gene expression programs accompanying *trans*-differentiation of epidermal cells of *Vicia faba* cotyledons into transfer cells. New Phytol. 182, 863–877. 10.1111/j.1469-8137.2009.02822.x 19383101

[B20] Endo H. Yamaguchi M. Tamura T. Nakano Y. Nishikubo N. Yoneda A.. (2015). Multiple classes of transcription factors regulate the expression of *VASCULAR-RELATED NAC-DOMAIN7*, a master switch of xylem vessel differentiation. Plant Cell Physiol. 56, 242–254. 10.1093/pcp/pcu134 25265867

[B21] Endo M. Shimizu H. Nohales M. A. Araki T. Kay S. A. (2014). Tissue-specific clocks in Arabidopsis show asymmetric coupling. Nature 515, 419–422. 10.1038/nature13919 25363766 PMC4270698

[B22] Felsenstein J. (1989). PHYLIP-phylogeny inference package (version 3.2). Cladistics 5, 164–166.

[B23] Fornalé S. Shi X. Chai C. Encina A. Irar S. Capellades M. (2010). ZmMYB31 directly represses maize lignin genes and directs the phenylpropanoid metabolic flux. Plant J. 64, 633–644. 10.1111/j.1365-313X.2010.04363.x 21070416

[B24] Fujita M. Fujita Y. Maruyama K. Seki M. Hiratsu K. Ohme-Takagi M. (2004). A dehydration-induced NAC protein, RD26, is involved in a novel ABA-dependent stress-induced signaling pathway. Plant J. 39, 863–876. 10.1111/j.1365-313X.2004.02171.x 15341629

[B25] Gottwald J. R. Krysan P. J. Young J. C. Evert R. F. Sussman M. R. (2000). Genetic evidence for the *in planta* role of phloem-specific plasma membrane sucrose transporters. Proc. Natl. Acad. Sci. U.S.A. 97, 13979–13984. 10.1073/pnas.250473797 11087840 PMC17686

[B26] Gunning B. E. Pate J. S. Briarty L. G. (1968). Specialized “transfer cells” in minor veins of leaves and their possible significance in phloem translocation. J. Cell Biol. 37, C7–C12. 10.1083/jcb.37.3.C7 11905215 PMC2107441

[B27] Gunning B. E. S. Pate J. S. (1974). Transfer cells, in Dynamic Aspects of Plant Ultrastructure, ed Robards A. W. (London: McGraw-Hill), 441–479.

[B28] Haritatos E. Medville R. Turgeon R. (2000). Minor vein structure and sugar transport in *Arabidopsis thaliana*. Planta 211, 105–111. 10.1007/s004250000268 10923710

[B29] Harrington G. Franceschi V. Offler C. Patrick J. Tegeder M. Frommer W. (1997). Cell specific expression of three genes involved in plasma membrane sucrose transport in developing *Vicia faba* seed. Protoplasma 197, 160–173. 10.1007/BF01288025

[B30] Haughn G. W. Western T. L. (2012). Arabidopsis seed coat mucilage is a specialized cell wall that can be used as a model for genetic analysis of plant cell wall structure and function. Front. Plant Sci. 3:64. 10.3389/fpls.2012.00064 22645594 PMC3355795

[B31] Hickman R. Hill C. Penfold C. A. Breeze E. Bowden L. Moore J. D.. (2013). A local regulatory network aound three NAC transcription factors in stress responses and senescence in Arabidopsis leaves. Plant J. 75, 26–39. 10.1111/tpj.12194 23578292 PMC3781708

[B32] Imlau A. Truernit E. Sauer N. (1999). Cell-to-cell and long-distance trafficking of the green fluorescent protein in the phloem and symplastic unloading of the protein into sink tissues. Plant Cell 11, 309–322. 10.1105/tpc.11.3.309 10072393 PMC144181

[B33] Jensen M. K. Kjaersgaard T. Nielsen M. M. Galberg P. Petersen K. O'Shea C.. (2010). The *Arabidopsis thaliana* NAC transcription factor family: structure-function relationships and determinants of ANAC019 stress signalling. Biochem. J. 426, 183–196. 10.1042/BJ20091234 19995345

[B34] Jin J. He K. Tang X. Li Z. Lv L. Zhao Y.. (2015). An Arabidopsis transcriptional regulatory map reveals distinct functional and evolutionary features of novel transcription factors. Molec. Biol. Evol. 32, 1767–1773. 10.1093/molbev/msv058 25750178 PMC4476157

[B35] Jin J. Tian F. Yang D. C. Meng Y. Q. Kong L. Luo J.. (2017). PlantTFDB 4.0: toward a central hub for transcription factors and regulatory interactions in plants. Nucl. Acid. Res. 45, D1040–D1045. 10.1093/nar/gkw982 27924042 PMC5210657

[B36] Kaplan D. R. Cooke T. J. (1997). Fundamental concepts in the embryogenesis of dicotyledons: a morphological interpretation of embryo mutants. Plant Cell 9, 1903–1919. 10.1105/tpc.9.11.1903 12237352 PMC157046

[B37] Kerstetter R. A. Poethig R. S. (1998). The specification of leaf identity during shoot development. Annu. Rev. Cell Dev. Biol. 14, 373–398. 10.1146/annurev.cellbio.14.1.373 9891788

[B38] Kircher S. Schopfer P. (2012). Photosynthetic sucrose acts as cotyledon-derived long-distance signal to control root growth during early seedling development in Arabidopsis. Proc. Natl. Acad. Sci. U.S.A. 109, 11217–11221. 10.1073/pnas.1203746109 22733756 PMC3396492

[B39] Kunieda T. Mitsuda N. Ohme-Takagi M. Takeda S. Aida M. Tasaka M.. (2008). NAC family proteins NARS1/NAC2 and NARS2/NAM in the outer integument regulate embryogenesis in Arabidopsis. Plant Cell 20, 2631–2642. 10.1105/tpc.108.060160 18849494 PMC2590734

[B40] Legay S. Sivadon P. Blervacq A. S. Pavy N. Baghdady A. Tremblay L.. (2010). EgMYB1, an R2R3 MYB transcription factor from eucalyptus negatively regulates secondary cell wall formation in Arabidopsis and poplar. New Phytol. 188, 774–786. 10.1111/j.1469-8137.2010.03432.x 20955415

[B41] Maeda H. Song W. Sage T. L. DellaPenna D. (2006). Tocopherols play a crucial role in low-temperature adaptation and phloem loading in Arabidopsis. Plant Cell 18, 2710–2732. 10.1105/tpc.105.039404 17012603 PMC1626601

[B42] Maeda H. Song W. Sage T. Dellapenna D. (2014). Role of callose synthases in transfer cell wall development in tocopherol deficient Arabidopsis mutants. Front. Plant Sci. 5:46. 10.3389/fpls.2014.00046 24600460 PMC3928550

[B43] Mahmood K. El-Kereamy A. Kim S. H. Nambara E. Rothstein S. J. (2016). ANAC032 positively regulates age-dependent and stress-induced senescence in *Arabidopsis thaliana*. Plant Cell Physiol. 57, 2029–2046. 10.1093/pcp/pcw120 27388337

[B44] Matiolli C. C. Tomaz J. P. Duarte G. T. Prado F. M. Del Bem L. E. Silveira A. B.. (2011). The Arabidopsis bZIP gene *AtbZIP63* is a sensitive integrator of transient abscisic acid and glucose signals. Plant Physiol. 157, 692–705. 10.1104/pp.111.181743 21844310 PMC3192551

[B45] McCurdy D. W. Patrick J. W. Offler C. E. (2008). Wall ingrowth formation in transfer cells: novel examples of localized wall deposition in plant cells. Curr. Opin. Plant Biol. 11, 653–661. 10.1016/j.pbi.2008.08.005 18849189

[B46] Nakano Y. Yamaguchi M. Endo H. Rejab N. A. Ohtani M. (2015). NAC-MYB-based transcriptional regulation of secondary cell wall biosynthesis in land plants. Front. Plant Sci. 6:288. 10.3389/fpls.2015.00288 25999964 PMC4419676

[B47] Nguyen S. T. Greaves T. McCurdy D. W. (2017). Heteroblastic development of transfer cells is controlled by the microRNA miR156/SPL module. Plant Physiol. 173, 1676–1691. 10.1104/pp.16.01741 28082719 PMC5338675

[B48] Nguyen S. T. McCurdy D. W. (2015). High-resolution confocal imaging of wall ingrowth deposition in plant transfer cells: semi-quantitative analysis of phloem parenchyma transfer cell development in leaf minor veins of Arabidopsis. BMC Plant Biol. 15, 109. 10.1186/s12870-015-0483-8 25899055 PMC4416241

[B49] Offler C. E. McCurdy D. W. Patrick J. W. Talbot M. J. (2003). Transfer cells: cells specialized for a special purpose. Annu. Rev. Plant Biol. 54, 431–454. 10.1146/annurev.arplant.54.031902.134812 14502998

[B50] Robinson M. D. McCarthy D. J. Smyth G. K. (2010). edgeR: a bioconductor package for differential expression analysis of digital gene expression data. Bioinform 26, 139–140. 10.1093/bioinformatics/btp616 19910308 PMC2796818

[B51] Robinson M. D. Smyth G. K. (2008). Small-sample estimation of negative binomial dispersion, with applications to SAGE data. Biostat 9, 321–332. 10.1093/biostatistics/kxm030 17728317

[B52] Robinson-Beers K. Sharkey T. D. Evert R. F. (1990). Import of ^14^C-photosynthate by developing leaves of sugarcane. Botan. Acta 103, 424–429. 10.1111/j.1438-8677.1990.tb00184.x

[B53] Rodiuc N. Vieira P. Banora M. Y. de Almeida Engler J. (2014). On the track of transfer cell formation by specialized plant-parasitic nematodes. Front. Plant Sci. 5:160. 10.3389/fpls.2014.00160 24847336 PMC4017147

[B54] Schweizer F. Bodenhausen N. Lassueur S. Masclaux F. G. Reymond P. (2013). Differential contribution of transcription factors to *Arabidopsis thaliana* defense against *Spodoptera littoralis*. Front. Plant Sci. 4:13. 10.3389/fpls.2013.00013 23382734 PMC3563046

[B55] Sha Y. Phan J. H. Wang M. D. (2015). Effect of low-expression gene filtering on detection of differentially expressed genes in RNA-seq data. Conf. Proc. IEEE Eng. Med. Biol. Soc. 2015, 6461–6464. 10.1109/EMBC.2015.7319872 26737772 PMC4983442

[B56] Shen H. He X. Poovaiah C. R. Wuddineh W. A. Ma J. Mann D. G.. (2012). Functional characterization of the switchgrass (*Panicum virgatum*) R2R3-MYB transcription factor PvMYB4 for improvement of lignocellulosic feedstocks. New Phytol. 193, 121–136. 10.1111/j.1469-8137.2011.03922.x 21988539

[B57] Sieburth L. E. Deyholos M. K. (2006). Vascular development: the long and winding road. Curr. Opin. Plant Biol. 9, 48–54. 10.1016/j.pbi.2005.11.008 16332447

[B58] Sievers F. Wilm A. Dineen D. Gibson T. J. Karplus K. Li W.. (2011). Fast, scalable generation of high-quality protein multiple sequence alignments using Clustal Omega. Mol. Syst. Biol. 7, 539. 10.1038/msb.2011.75 21988835 PMC3261699

[B59] Simmons A. R. Bergmann D. C. (2016). Transcriptional control of cell fate in the stomatal lineage. Curr. Opin. Plant Biol. 29, 1–8. 10.1016/j.pbi.2015.09.008 26550955 PMC4753106

[B60] Sonbol F. M. Fornalé S. Capellades M. Encina A. Touriño S. Torres J. L.. (2009). The maize ZmMYB42 represses the phenylpropanoid pathway and affects the cell wall structure, composition and degradability in *Arabidopsis thaliana*. Plant Mol. Biol. 70, 283–296. 10.1007/s11103-009-9473-2 19238561

[B61] Soneson C. Delorenzi M. (2013). A comparison of methods for differential expression analysis of RNA-seq data. BMB Bioinformatics 14:91. 10.1186/1471-2105-14-91 23497356 PMC3608160

[B62] Symonds V. V. Hatlestad G. Lloyd A. M. (2011). Natural allelic variation defines a role for *ATMYC1*: trichome cell fate determination. PLoS Genet. 7:e1002069. 10.1371/journal.pgen.1002069 21695236 PMC3111535

[B63] Szakasits D. Heinen P. Wieczorek K. Hofmann J. Wagner F. Kreil D. P.. (2009). The transcriptome of synctia induced by the cyst nematode *Heterodera schachtii* in Arabidopsis roots. Plant J. 57, 771–784. 10.1111/j.1365-313X.2008.03727.x 18980640 PMC2667683

[B64] Taylor-Teeples M. Lin L. de Lucas M. Turco G. Toal T. W. Gaudinier A.. (2015). An Arabidopsis gene regulatory network for secondary cell wall synthesis. Nature 517, 571–575. 10.1038/nature14099 25533953 PMC4333722

[B65] Thiel J. (2014). Development of endosperm transfer cells in barley. Front. Plant Sci. 5:108. 10.3389/fpls.2014.00108 24723929 PMC3972472

[B66] Thiel J. Riewe D. Rutten T. Melzer M. Friedel S. Bollenbeck F.. (2012). Differentiation of endosperm transfer cells of barley: a comprehensive analysis at the micro-scale. Plant J. 71, 639–655. 10.1111/j.1365-313X.2012.05018.x 22487146

[B67] Thiel J. Weier D. Sreenivasulu N. Strickert M. Weichert N. Melzer M.. (2008). Different hormonal regulation of cellular differentiation and function in nucellar projection and endosperm transfer cells: a microdissection-based transcriptome study of young barley grains. Plant Physiol. 148, 1436–1452. 10.1104/pp.108.127001 18784282 PMC2577268

[B68] Toledo-Ortiz G. Huq E. Quail P. H. (2003). The Arabidopsis basic/helix-loop-helix transcription factor family. Plant Cell 15, 1749–1770. 10.1105/tpc.013839 12897250 PMC167167

[B69] Tran L. S. Nakashima K. Sakuma Y. Simpson S. D. Fujita Y. Maruyama K.. (2004). Isolation and functional analysis of Arabidopsis stress-inducible NAC transcription factors that bind to a drought-responsive cis-element in the early responsive to dehydration stress 1 promoter. Plant Cell 16, 2481–2498. 10.1105/tpc.104.022699 15319476 PMC520947

[B70] Tsai A. Y. Kunieda T. Rogalski J. Foster L. J. Ellis B. E. Haughn G. W. (2017). Identification and characterization of Arabidopsis seed coat mucilage proteins. Plant Physiol. 173, 1059–1074. 10.1104/pp.16.01600 28003327 PMC5291037

[B71] Tsukaya H. Shoda K. Kim G. T. Uchimiya H. (2000). Heteroblasty in *Arabidopsis thaliana* (L.) Heynh. Planta 210, 536–542. 10.1007/s004250050042 10787046

[B72] Van Lijsebettens M. Clarke J. (1998). Leaf development in Arabidopsis. Plant Physiol. Biochem. 36, 47–60. 10.1016/S0981-9428(98)80090-9

[B73] Vaughn K. C. Talbot M. J. Offler C. E. McCurdy D. W. (2007). Wall Ingrowths in epidermal transfer cells of *Vicia faba* cotyledons are modified primary walls marked by localized accumulations of arabinogalactan proteins. Plant Cell Physiol. 48, 159–168. 10.1093/pcp/pcl047 17169921

[B74] Vermeirssen V. De Clercq I. Van Parys T. Van Breusegem F. Van de Peer Y. (2014). Arabidopsis ensemble reverse-engineered gene regulatory network discloses interconnected transcription factors in oxidative stress. Plant Cell 26, 4656–4679. 10.1105/tpc.114.131417 25549671 PMC4311199

[B75] Voiniciuc C. Schmidt M. H. Berger A. Yang B. Ebert B. Scheller H. V.. (2015). MUCILAGE-RELATED10 produces galactoglucomannan that maintains pectin and cellulose architecture in Arabidopsis seed mucilage. Plant Physiol. 169, 403–420. 10.1104/pp.15.00851 26220953 PMC4577422

[B76] Wang S. Yin Y. Ma Q. Tang X. Hao D. Xu Y. (2012). Genome-scale identification of cell-wall related genes in Arabidopsis based on co-expression network analysis. BMC Plant Biol. 12:138. 10.1186/1471-2229-12-138 22877077 PMC3463447

[B77] Wu Y. (2017). Investigating Transcriptional Regulation of Transfer Cell Development in Arabidopsis Thaliana. Ph.D. thesis, University of Newcastle, Newcastle, NSW.

[B78] Wu Y. Deng Z. Lai J. Zhang Y. Yang C. Yin B.. (2009). Dual function of Arabidopsis ATAF1 in abiotic and biotic stress responses. Cell Res. 19, 1279–1290. 10.1038/cr.2009.108 19752887

[B79] Xiong Y. Li Q. B. Kang B. H. Chourey P. S. (2011). Discovery of genes expressed in basal endosperm transfer cells in maize using 454 transcriptome sequencing. Plant Mol. Biol. Rep. 29, 835–847. 10.1007/s11105-011-0291-8

[B80] Yamaguchi M. Ohtani M. Mitsuda N. Kubo M. Ohme-Takagi M. Fukuda H.. (2010). VND-INTERACTING2, a NAC domain transcription factor, negatively regulates xylem vessel formation in Arabidopsis. Plant Cell 22, 1249–1263. 10.1105/tpc.108.064048 20388856 PMC2879754

[B81] Zhang H. M. Wheeler S. Xia X. Radchuk R. Weber H. Offler C. E.. (2015). Differential transcriptional networks associated with key phases of ingrowth wall construction in *trans*-differentiating epidermal transfer cells of *Vicia faba* cotyledons. BMC Plant Biol. 15, 103. 10.1186/s12870-015-0486-5 25887034 PMC4437447

[B82] Zhao C. Avci U. Grant E. H. Haigler C. H. Beers E. P. (2008). XND1, a member of the NAC domain family in Arabidopsis thaliana, negatively regulates lignocellulose synthesis and programmed cell death in xylem. Plant J. 53, 425–436. 10.1111/j.1365-313X.2007.03350.x 18069942

[B83] Zheng X. Y. Spivey N. W. Zeng W. Liu P. P. Fu Z. Q. Klessig D. F.. (2012). Coronatine promotes *Pseudomonas syringae* virulence in plants by activating a signaling cascade that inhibits salicylic acid accumulation. Cell Host Microbe 11, 587–596. 10.1016/j.chom.2012.04.014 22704619 PMC3404825

[B84] Zhong R. Ye Z. H. (2015). Secondary cell walls: biosynthesis, patterned deposition and transcriptional regulation. Plant Cell Physiol. 56, 195–214. 10.1093/pcp/pcu140 25294860

[B85] Zhou Y. Andriunas F. Offler C. E. McCurdy D. W. Patrick J. W. (2010). An epidermal-specific ethylene signal cascade regulates trans-differentiation of transfer cells in *Vicia faba* cotyledons. New Phytol. 185, 931–943. 10.1111/j.1469-8137.2009.03136.x 20085619

