# Supplementary material for: Transcript Profiling Identifies NAC-Domain Genes Involved in Regulating Wall Ingrowth Deposition in Phloem Parenchyma Transfer Cells of Arabidopsis thaliana
Source: Front Plant Sci. 2018 Mar 15;9:341. doi: 10.3389/fpls.2018.00341 (PMC5862824; doi:10.3389/fpls.2018.00341)
Supplement: Supplementary file 1 [file DataSheet1.PDF]

Supplementary Material

Transcript profiling identifies NAC-domain genes involved in regulating wall ingrowth deposition in phloem parenchyma transfer cells of *Arabidopsis thaliana*

Yuzhou Wu, Jiexi Hou, Fen Yu, Suong T.T. Nguyen, David W. McCurdy*

*** Correspondence:** David W. McCurdy: David.McCurdy@newcastle.edu.au

1 MATERIALS and METHODS

1.1 Extraction of genomic DNA and PCR-based genotyping of Arabidopsis T-DNA insertional mutants

Extraction of genomic DNA for genotyping of Arabidopsis lines was conducted based on the rapid procedures in Berendzen et al. (2005). Briefly, a single cotyledon from each 10-day-old seedling grown on MS agar plates was cut and transferred to a labelled microfuge tube under sterile conditions, which was then immediately snap frozen in liquid nitrogen. After all samples from a given genotyping experiment were collected, the tissue pieces were ground using sterile micropestles for 30 seconds, then ground for another 30 seconds after adding 50-100 μL DNA extraction buffer containing 50 mM Tris-HCl (pH 7.5), 300 mM NaCl and 300 mM sucrose. The ground sample was then immediately stored on ice and after a group of samples were collected within 10 minutes, they were then incubated at 100°C for 10 minutes, and followed by centrifugation for 10 seconds at 6000 g. Thereafter, the extracted genomic DNA was either used directly for subsequent PCR-based genotyping, or stored at -20°C for longer term.

To identify homozygous individuals for Arabidopsis T-DNA insertional mutants, genotyping was performed based on a PCR strategy using two sets of primers: “control” primer set made up of left and right primers (LP+RP) that amplifies an approximately 1 kb fragment of the coding sequence in wild-type (WT) and surrounding the T-DNA insertion in each relevant mutant line, while the other “mutant” primer set used the right primer (RP) in combination with a left border (LB) primer on the T-DNA insertion. Details for structure of genes, location of insertions and primers as well as the determination of genotypes is presented in Supplementary Figure S4.

For each individual insertional mutant line, usually genomic DNA from eight different seedlings plus one WT seedling as control were used in PCR for genotyping of insertional mutants. One μL of each genomic DNA was added to a mix of 0.5 μL each of 10 μM primer stock solutions, 0.125 μL of the Taq DNA Polymerase plus 2.5 μL 10X Standard Taq Reaction Buffer (NEB, M0273) and 20.9 μL nuclease-free MQ water to make 25 μL final volume. The reactions using both “control” and “mutant” primer sets for all mutants to be genotyped were carried out in Eppendorf thermal cycler (Mastercycler personal and/or Mastercycler ep gradient S) programmed as follows: Initial denaturation at 95°C for

30 seconds, denaturation at 95°C for 30 seconds, annealing at 55°C for 30 seconds, extension at 68°C for 60 seconds, and after 40 repeated cycles from the denaturation to extensions, there was a final extension at 68°C for 5 minutes.

Subsequently, each 25µL PCR product was mixed with 5 µL of Fermentas 6x Loading Buffer and 20 µL of this volume was loaded into each lane of 1% (w/v) agarose gel containing 0.1% (w/v) ethidium bromide, along with 5 µL of GeneRuler 1 kb added to a single lane of each gel. After electrophoresis at 110 V, gels were photographed using GelDoc XR Imaging System (BIORAD) and Quantity One Ver. 4.6.6 software.

1.2 Semi-quantitative RT-PCR

WT and homozygous plants were transferred from MS agar plates into soil after 7-10 days, and mature juvenile Leaf 1 and Leaf 2 from these plants at 3-w old were collected into labelled RNase-free microfuge tubes and immediately snap-frozen in liquid nitrogen. The RNA extraction, on-column gDNA elimination and assessment of RNA yield and purity was conducted as described in Materials and Methods in the main body.

Reverse transcription of first strand cDNA synthesis was performed using 1 µg of total RNA extracted as described above and Superscript III™ First-Strand Synthesis System (Invitrogen) following the manufacturer's instructions. Subsequently, 1 µL cDNA products of each reverse transcription reactions were used as template in 25 µL PCR reactions using forward and reverse primers (labelled as For and Rev in green, see Supplementary Figure S4) designed for genes with T-DNA insertions plus *ACT2* (*AT3G18780*) as housekeeping control and NEB's Taq DNA Polymerase with Standard Taq Buffer (M0273) as described in the above section. Semi-quantification of gene expression was achieved by using the Gel tool in *ImageJ* that measures the intensity of bands on gels, and then the change of expression was normalized to the expression level of *ACT2* gene.

1.3 References

Berendzen, K., Searle, I., Ravenscroft, D., Koncz, C., Batschauer, A., Coupland, G., et al. (2005). A rapid and versatile combined DNA/RNA extraction protocol and its application to the analysis of a novel DNA marker set polymorphic between *Arabidopsis thaliana* ecotypes Col-0 and Landsberg *erecta*. *Plant Methods* 1**,** 4. doi: 10.1186/1746-4811-1-4

2. Supplementary Figures and Tables

2.1 Supplementary Figures

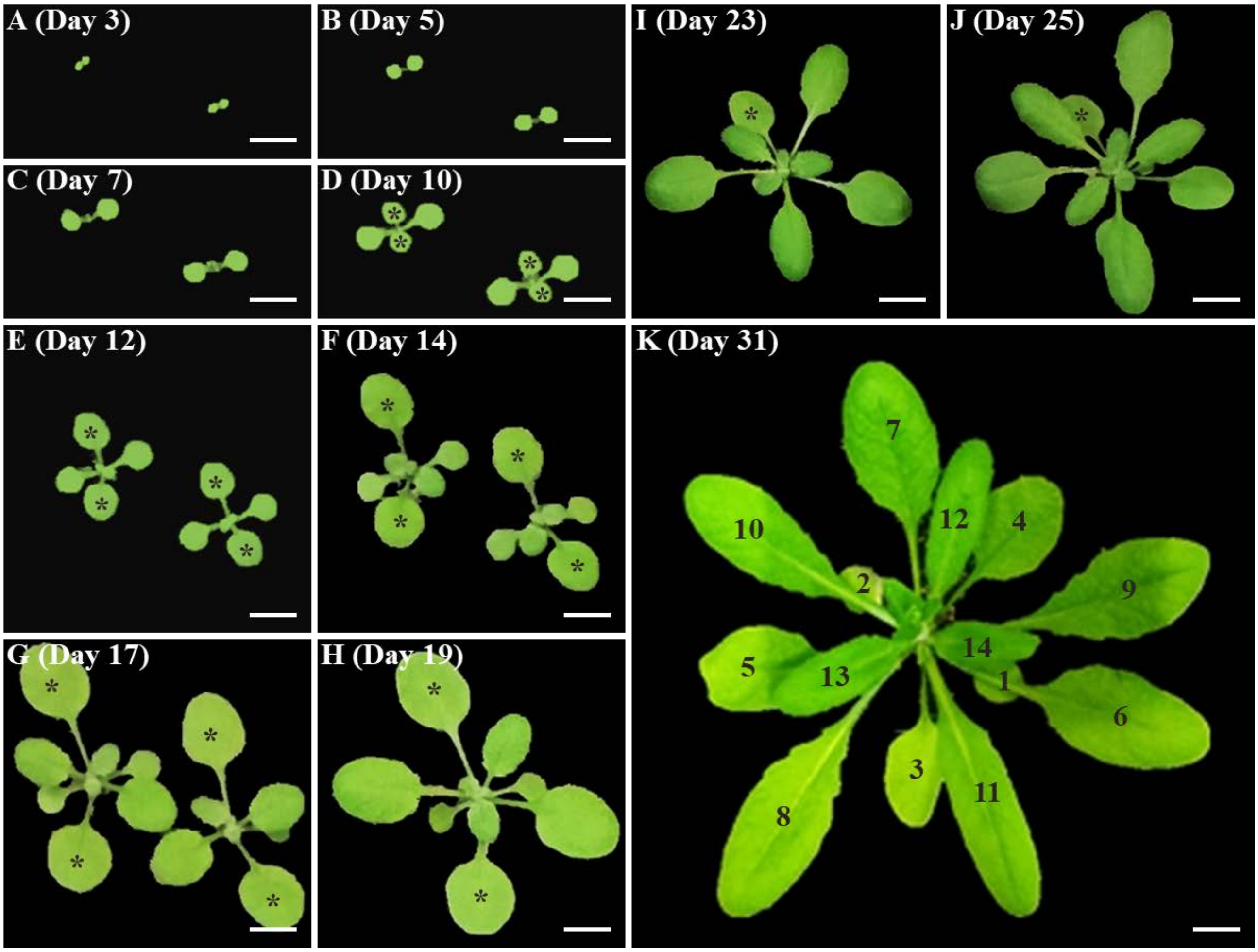

Supplementary Figure 1. Growth of cotyledons and leaves of Arabidopsis plants grown in soil. (A–K) Growth at Day 3, 5, 7, 10, 12, 14, 17, 19, 23, 25 and 31, respectively. Cotyledons were visible at Day 3 **(A)**, while Leaf 1 and Leaf 2 were visible by Day 7 **(C)**. At Day 12 **(E)**, Leaf 1 and Leaf 2 (asterisks) have expanded to exceed the size of the cotyledons. A total of 4, 5, 7, 8, 10, 12 and 14 rosette leaves visible at Day 12, 14, 17, 19, 23, 25 and 31, respectively, all showing the typical clockwise/anticlockwise spiral with constant divergence angle between successive leaves **(E–K)**. Asterisks, first pair rosette leaves. Scale bars = 5 mm (**A–H**); 10 mm (**I–K**).

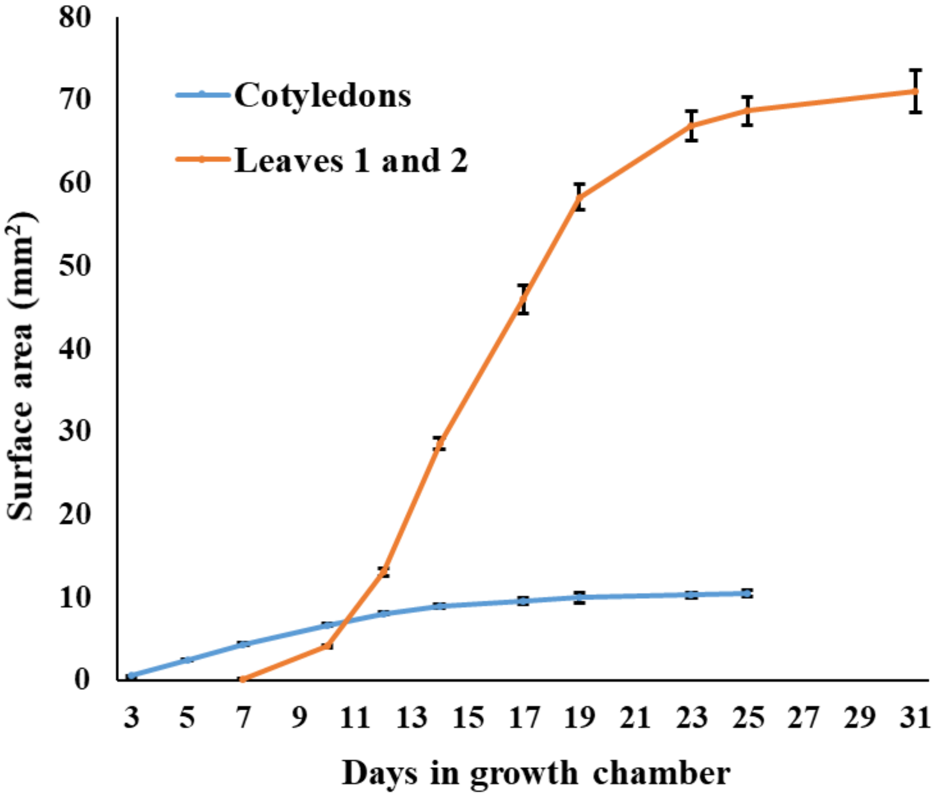

Supplementary Figure 2. Quantitative analysis of surface area expansion of cotyledons and Leaf 1 and 2 across Arabidopsis rosette development. Cotyledon surface area expanded in an apparent curvilinear manner with maximum expansion occurring from Day 3 to Day 10 and plateauing by approximately Day 17 (blue line). Leaf 1 and 2 surface areas expanded in a sigmoidal manner with linear expansion occurring from Day 10 to Day 19 and reaching maximum surface area (fully expanded) by approximately Day 25. Data shows mean ± SE for surface area (mm^2); plateauing stages were statistically determined by one-way ANOVA applied *a priori* contrasts and t-test thereafter; *n* >3 in all cases.

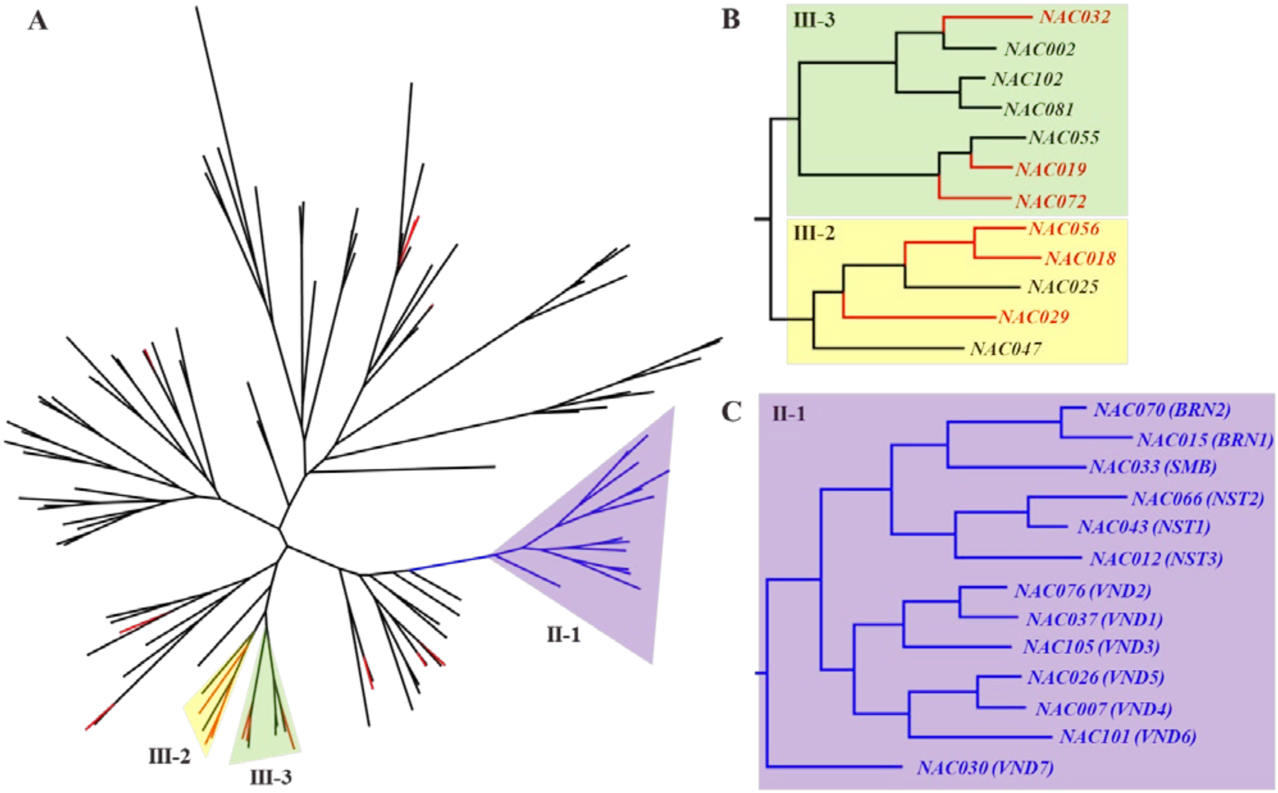

Supplementary Figure 3. Phylogenetic tree of NAC-domain transcription factors in Arabidopsis. (A) Radial tree layout. Red lines indicate the 15 NACs identified as commonly up-regulated in at least two of the three experimental conditions comparing wall ingrowth abundance from RNA-Seq analysis reported in Table 2 and Table S1. Of these 15 NACs, three belonged to Clade III-2 (yellow shading) while another three belonged to Clade III-3 (green shading). Blue lines indicate 10 NACs associated with secondary wall deposition belonging to Clade II-1 (purple shading). **(B)** Enlarged view of Clade III-3 (green shading) and Clade III-2 (yellow shading) with the individual NACs emerging from the RNA-Seq analysis (Table 2 and Table S1) shown in red. **(C)** Enlarged view of Clade II-1 (purple shading) with the individual secondary wall NAC master switches shown in blue (*VND1*-*VND7*, *NST1*-*NST3*, *SMB*, *BRN1* and *BRN2*).

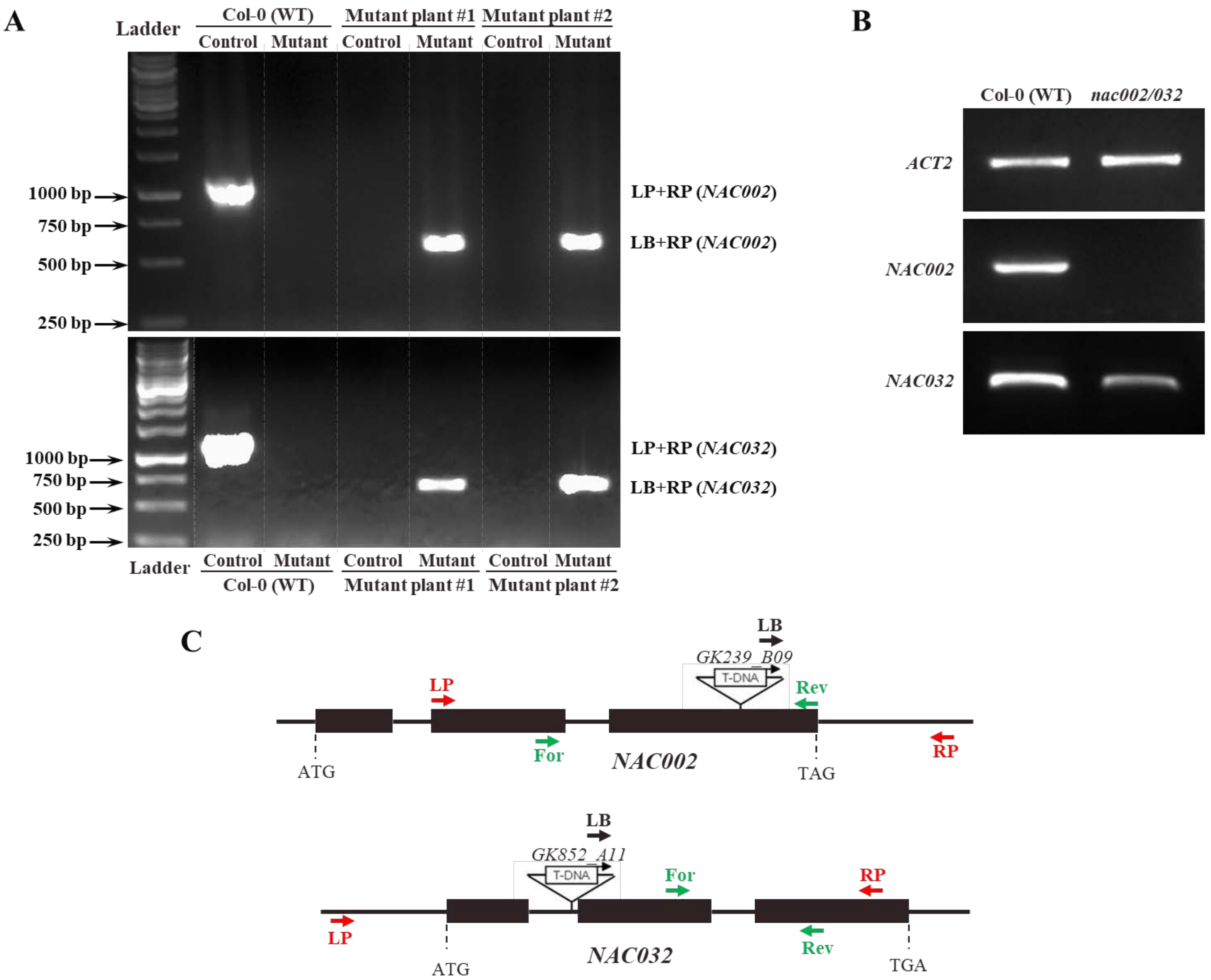

Supplementary Figure 4. Molecular analysis of *nac002/032* double mutant obtained from the GABI-DUPLO collection. (A) Examples of two homozygous plants identified by PCR genotyping. Top gel: Genotyping results for insertion *GK_239B09* carried in *NAC002*. A band of approx. 1000 bp was amplified from Col-0 (WT) using "Control PCR" (LP+RP primers), but not by using "Mutant PCR" (LB+RP). In contrast, genomic DNA isolated from two mutant plant lines #1 and #2 supported amplification of an approximately 500 bp fragment using the "Mutant PCR" (LB+RP) but not "Control PCR" (LP+RP). No 1000 bp band was detected in these mutant lines using the LP+RP primer set, indicating that the two individual plants #1 and #2 were homozygous for insertion *GK_239B09* in *NAC002*. Bottom gel: Genotyping results for insertion *GK_852A11* carried in *NAC032*. A band of approx. 1000 bp was amplified from Col-0 (WT) using "Control PCR" (LP+RP primers), but not using "Mutant PCR" (LB+RP). In contrast, genomic DNA isolated from two mutant plant lines #1 and #2 supported amplification of an approx. 700 bp fragment using the "Mutant PCR" (LB+RP) but not "Control PCR" (LP+RP). No 1000 bp band was detected in these mutant lines using the LP+RP primer set, indicating that the same two individual plants #1 and #2 were homozygous for insertion *GK_852A11* in *NAC032*. DNA ladder is shown to the left in both gels. **(B)** RT-PCR analysis of *NAC002* and *NAC032* expression in juvenile leaves of WT and the double homozygous line *nac002/032*. Expression of *NAC002* and *NAC032* is clearly evident in Col-0 (WT), but message of *NAC002* in the *nac002/nac032* double mutant is absent. In contrast, *NAC032* expression in the double mutant is knocked down by about 60%. *ACT2* was used as a loading

control. **(C)** Structure of T-DNA insertional mutants of *NAC002* and *NAC032*. T-DNA insertion of *GK_239B09* is located in the last exon (black box) of *NAC002*, while *GK_852A11* is located in the first intron (black line between boxes) of *NAC032*. Primer sets of LP and RP (red) were used for amplification of Col-0 (WT) gene flanking the insertions, while LB primer (black) is located in the T-DNA insertion. Primer sets For and Rev (green) were used for RT-PCR analysis of gene expression in the relevant insertional mutant line. ATG = start of coding region; TAG/TGA = stop codon.

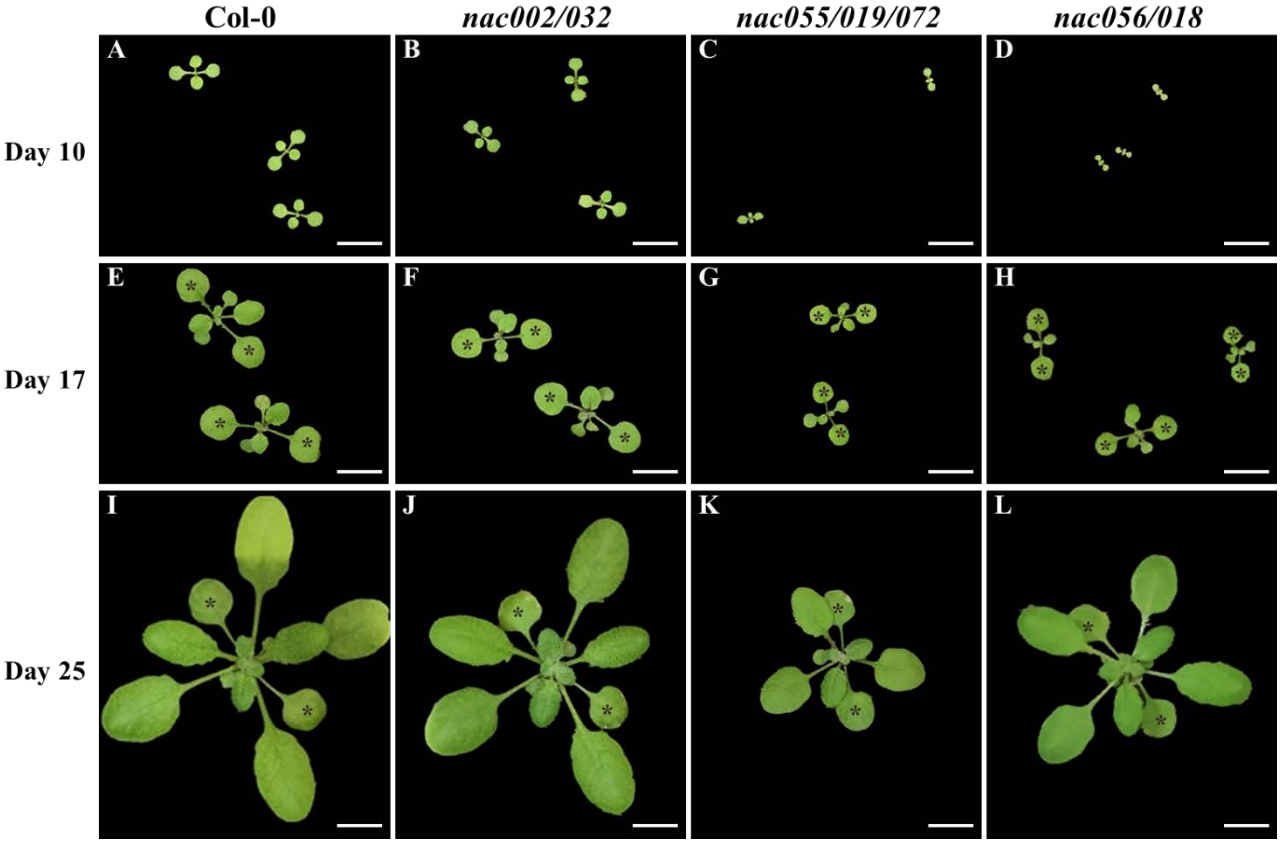

Supplementary Figure 5. Growth of cotyledons and leaves of Col-0 and *nac* mutant plants grown in soil. **(A–D)** By Day 10, all WT and mutants developed a pair of cotyledons and first true Leaf 1 and 2, but sizes of *nac055/019/072* **(C)** and *nac056/018* **(D)** were much smaller than Col-0 **(A)** and *nac002/032* **(B)**. **(E–F)** By Day 17, 5-6 rosette leaves were visible in Col-0 **(E)** and *nac002/032* **(F)** while only 3-4 leaves had emerged in *nac055/019/072* **(G)** and *nac056/018* **(H)**. **(I–L)** By Day 25, rosette size was similar between Col-0 **(I)** and *nac002/032* **(J)** but both *nac055/019/072* **(K)** and *nac056/018* **(L)** were smaller; note that the size of the two first leaves were comparable between Col-0 and *nac002/032*, but these leaves were clearly smaller for *nac055/019/072* and *nac056/018*. Asterisks, first pair rosette leaves. Scale bars = 10 mm in all panels.

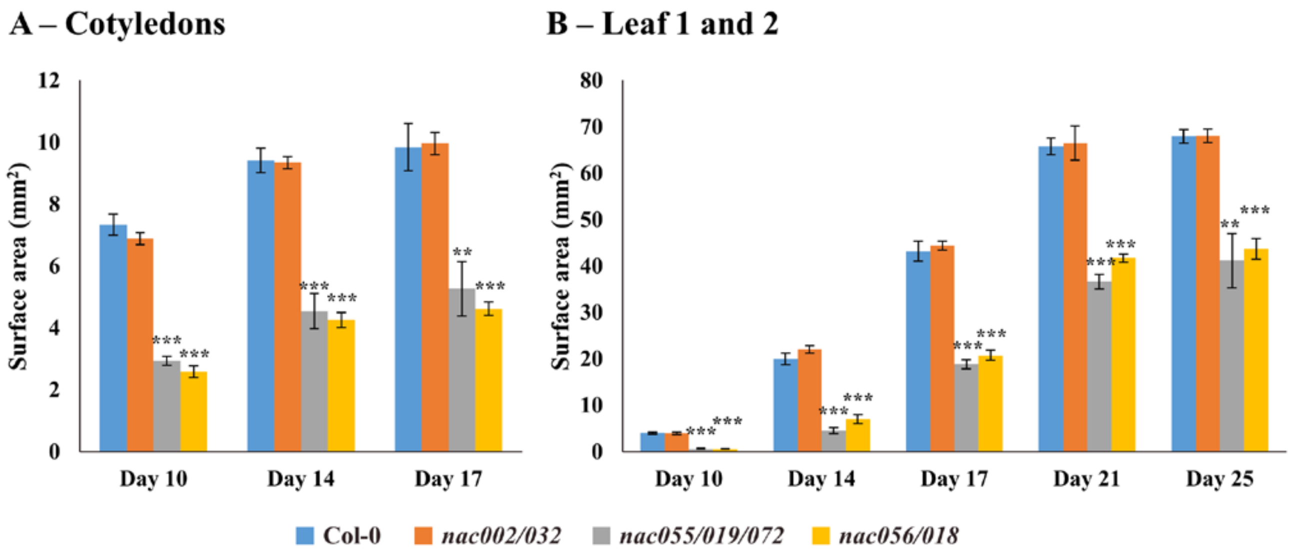

Supplementary Figure 6. Quantitative analysis of surface area expansion of cotyledons and first pair leaves across rosette development in Col-0 and *nac* mutant plants. (A) Cotyledon surface area expanded from Day 10 to Day 14 and reaching maximum surface area (fully expanded) by Day 17, in Col-0 and all *nac* mutants. **(B)** Leaf 1 and 2 surface area expanded rapidly from Day 10 to Day 21 and plateauing by Day 25. Surface area of cotyledons and first leaves in *nac055/019/072* and *nac056/018* were statistically smaller than in Col-0 at all tested stages. Data shows mean ± SE for surface area (mm^2). ***P*<0.01, ****P*<0.001, student's t-test comparing cotyledons and leaves size in each mutant line with Col-0 at each tested developmental stage, *n* = 3-5.

2.2 Supplementary Tables

Supplementary Table 1. Mapping statistics for each sample as reported from CLC Genomics Workbench

Samples	Replicate	Trimmed reads	Mapped reads	% [a]	Uniquely mapped	% [b]	Non-specifically mapped	% [c]
**Day 5 Cotyledons**	#1	36,764,981	36,210,704	98	34,846,403	96	1,364,301	4
	#2	35,699,447	35,101,316	98	33,747,545	96	1,353,771	4
	#3	45,334,263	44,690,500	99	43,043,513	96	1,646,987	4
**Day 10 Cotyledons**	#1	38,363,610	37,876,410	99	36,517,721	96	1,358,689	4
	#2	34,682,950	34,273,441	99	33,051,324	96	1,222,117	4
	#3	40,402,450	39,903,809	99	38,447,328	96	1,456,481	4
**Day 10 First leaves**	#1	40,132,730	39,618,789	99	38,183,958	96	1,434,831	4
	#2	40,729,962	40,173,683	99	38,732,196	96	1,441,487	4
	#3	41,400,529	40,927,734	99	39,602,921	97	1,324,813	3
**Day 16 First leaves**	#1	39,687,664	39,223,692	99	37,841,449	96	1,382,243	4
	#2	37,004,904	36,491,345	99	35,144,432	96	1,346,913	4
	#3	40,509,893	39,983,310	99	38,455,239	96	1,528,071	4
**Leaf 12 (Day 31) Basal**	#1	55,597,019	54,922,942	99	51,510,611	94	3,412,331	6
	#2	49,900,669	49,000,172	98	45,314,800	92	3,685,372	8
	#3	51,570,790	50,921,920	99	47,709,324	94	3,212,596	6
**Leaf 12 (Day 31) Apical**	#1	51,971,922	51,243,442	99	48,097,777	94	3,145,665	6
	#2	65,039,217	64,225,107	99	60,144,028	94	4,081,079	6
	#3	48,753,901	47,826,936	98	44,411,042	93	3,415,894	7

[a] Percentage of mapped reads among trimmed reads;
[b] Percentage of uniquely mapped reads among total mapped reads;
[c] Percentage of non-specifically mapped reads among total mapped reads.

Supplementary Table 2. Transcription factors (51 in total) showing differential up-regulation in two of the three experimental comparisons analyzing wall ingrowth deposition in PP TCs of Arabidopsis

Locus [a]	Symbol [b]	Family [c]	(i) Cotyledon Day 5 vs Day 10		(ii) First leaves Day 10 vs Day 16		(iii) Leaf 12 (Day 31) Basal vs apical	
			P [d]	**FC** [e]	***P***	**FC**	***P***	**FC**
AT4G01540	*NAC68*	NAC	0.00	2.7 [f]	0.00	7.3	0.04	1.8
AT4G27410	*NAC072*	NAC	0.24	-1.9	0.00	3.4	0.00	3.1
AT1G77450	*NAC032*	NAC	0.34	1.2	0.00	3.3	0.00	2.3
AT3G04420	*NAC048*	NAC	0.00	2.5	0.01	2.3	1.00	1.0
AT1G52890	*NAC019*	NAC	0.12	+∞ [g]	0.01	+∞	0.00	3.6
AT5G07680	*NAC080*	NAC	0.00	3.4	0.00	+∞	0.16	1.8
AT1G34180	*NAC016*	NAC	0.05	2.0	0.00	+∞	0.09	+∞
AT3G44350	*NAC061*	NAC	0.01	+∞	0.00	+∞	0.42	1.7
AT5G39610	*NAC092*	NAC	0.00	+∞	0.32	+∞	0.00	+∞
AT2G43000	*NAC042*	NAC	0.00	+∞	0.00	+∞	0.17	-∞
AT2G20880	*ERF053*	ERF	0.01	-1.6	0.00	3.7	0.00	5.9
AT5G25190	*ERF003*	ERF	0.00	6.4	1.00	-1.1	0.00	2.7
AT2G31230	*ERF15*	ERF	0.00	4.3	0.00	4.4	1.00	1.1
AT4G39780	*ERF060*	ERF	0.02	1.7	0.00	5.3	0.00	2.3
AT1G46768	*RAP2-1*	ERF	0.62	1.2	0.00	2.8	0.00	2.2
AT2G22200	*ERF056*	ERF	0.00	-1.7	0.00	2.9	0.00	2.1
AT1G72360	*ERF073*	ERF	0.00	2.1	0.00	2.8	0.17	1.5
AT4G36900	*RAP2-10*	ERF	1.00	1.0	0.00	2.3	0.00	2.3
AT4G34410	*ERF109*	ERF	0.00	+∞	0.28	-∞ [h]	0.00	+∞
AT4G05100	*AtMYB74*	MYB	1.00	1.0	0.00	13.0	0.00	3.9
AT3G11280	*AT3G11280*	MYB	0.00	2.0	0.00	6.8	0.00	1.4
AT5G07700	*MYB76*	MYB	0.00	3.2	0.00	4.3	1.00	1.0
AT5G61420	*MYB28*	MYB	0.00	2.8	0.00	3.2	0.23	1.2
AT5G07690	*MYB29*	MYB	0.00	2.8	0.00	3.1	0.18	-1.3
AT1G15790	*AT1G15790*	bHLH	0.00	4.3	0.00	10.9	0.22	1.5
AT3G07340	*BHLH62*	bHLH	0.00	3.3	0.00	2.0	0.62	1.2
AT2G46970	*PIL1*	bHLH	0.01	+∞	0.00	2.7	0.08	2.0
AT3G19500	*BHLH113*	bHLH	0.03	+∞	0.00	+∞	1.00	-1.1
AT5G52830	*WRKY27*	WRKY	0.14	1.3	0.00	8.8	0.00	2.5
AT2G25000	*WRKY60*	WRKY	0.00	2.4	0.00	3.9	0.31	1.5
AT5G07100	*WRKY26*	WRKY	0.00	6.1	0.00	+∞	0.93	-1.3
AT5G06510	*NFYA10*	NF-YA	0.38	-1.3	0.00	6.6	0.01	2.5
AT1G54160	*NFYA5*	NF-YA	0.00	-1.8	0.00	2.8	0.00	2.1
AT1G77920	*TGA7*	bZIP	0.00	2.2	0.00	6.4	0.01	1.5
AT2G46270	*GBF3*	bZIP	1.00	-1.0	0.00	2.9	0.00	2.4
AT5G10970	*AT5G10970*	C2H2	0.00	2.1	0.00	2.8	1.00	1.1
AT3G46090	*ZAT7*	C2H2	0.00	+∞	0.00	+∞	0.64	-1.4
AT1G07900	*LBD1*	LBD	0.03	-1.8	0.00	+∞	0.00	4.7
AT3G27940	*LBD26*	LBD	0.00	+∞	0.00	+∞	0.12	1.5
AT1G53160	*SPL4*	SBP	0.00	5.0	0.00	22.3	1.00	-1.1
AT2G18328	*RL4*	MYB-related	0.00	2.2	0.00	6.2	0.00	1.6
AT1G16060	*ADAP*	AP2	0.00	2.6	0.00	4.5	1.00	1.1
AT2G33550	*AT2G33550*	Trihelix	0.00	2.1	0.00	4.3	0.00	1.5
AT2G22430	*ATHB-6*	HD-ZIP	0.00	2.0	0.00	4.3	0.00	1.6
AT4G34680	*GATA3*	GATA	0.00	2.2	0.00	4.1	0.01	1.5
AT1G64620	*DOF1.8*	Dof	0.00	2.4	0.00	3.3	0.07	1.4
AT2G35940	*BLH1*	TALE	0.00	2.7	0.00	2.9	0.00	1.6
AT2G37650	*SCL9*	GRAS	0.00	2.0	0.00	2.7	0.06	1.4
AT1G04990	*AT1G04990*	C3H	0.00	2.2	0.00	2.1	1.00	1.1
AT4G18960	*AG*	MIKC MADS	NA [i]	NA	0.00	+∞	0.00	3.1

AT2G35550	*BPC7*	BBR-BPC	0.01	2.1	0.00	+∞	0.48	1.7

[a, b] Gene locus and symbol was retrieved from TAIR10.
[c] Transcription factors were classified into families based on the assignment rules in PlantTFDB. Within each family, transcription factors were ranked according to highest individual fold-change across the three experimental comparisons.
[d] *P*-value adjusted by FDR, calculated by *Empirical analysis of DGE* in CLC Genomics Workbench.
[e] FC - fold-change in expression of genes between data sets (comparing samples with abundant vs no wall ingrowths), calculated by *Empirical analysis of DGE* in CLC Genomics Workbench.
[f] Fold-changes of ≥2 or +∞ with *P* <0.05 are indicated in red.
[g] +∞ indicates gene was not expressed (exon counts <10) in samples with no detectable wall ingrowth, but was expressed (exon counts ≥ 10) in samples with abundant wall ingrowth deposition.
[h] -∞ indicates gene was not expressed (exon counts <10) in samples with abundant wall ingrowth deposition, but was expressed (exon counts ≥ 10) in samples with no detectable wall ingrowths.
[i] NA indicates that the gene was not expressed in both samples of experimental comparison (i), thus no available fold-change or *P*-value.

Supplementary Table 3. Transcription factors (100 in total) showing differential down-regulation in two of the three experimental comparisons analyzing wall ingrowth deposition in PP TCs of Arabidopsis.

Locus [a]	Symbol [b]	Family [c]	(i) Cotyledon Day 5 vs Day 10		(ii) First leaves Day 10 vs Day 16		(iii) Leaf 12 (Day 31) Basal vs apical	
			P [d]	FC [e]	*P*	FC	*P*	FC
AT5G26660	*MYB86*	MYB	NA [f]	NA	0.00	-15.9 [g]	0.00	-3.2
AT3G50060	*MYB77*	MYB	0.95	1.3	0.00	-3.5	0.00	-8.0
AT4G37260	*MYB73*	MYB	0.44	-1.7	0.00	-6.4	0.00	-4.0
AT5G11510	*MYB3R-4*	MYB	0.00	-2.7	0.00	-5.6	0.01	-1.6
AT5G12870	*MYB46*	MYB	0.00	-2.8	0.00	-3.5	0.25	-1.5
AT5G15310	*MYB16*	MYB	0.00	-2.7	0.00	-3.0	0.14	1.2
AT5G01200	*AT5G01200*	MYB	0.00	-3.0	0.00	-2.5	0.01	-1.9
AT4G01680	*MYB55*	MYB	0.00	-2.2	0.00	-2.2	0.40	-1.6
AT5G16600	*MYB43*	MYB	0.00	-2.3	0.00	-2.1	0.38	1.4
AT3G61250	*MYB17*	MYB	0.00	-∞ [h]	0.00	-5.1	1.00	-1.3
AT3G08500	*MYB83*	MYB	0.02	-2.1	0.00	-∞	0.13	-1.9
AT1G71930	*NAC030*	NAC	0.00	-2.4	0.00	-5.8	0.10	-1.7
AT5G62380	*NAC101*	NAC	0.00	-3.6	0.00	-4.4	0.08	-1.6
AT1G12260	*NAC007*	NAC	0.00	-2.5	0.00	-4.1	0.00	-1.8
AT5G50820	*NAC097*	NAC	0.00	-3.7	1.00	-1.1	0.00	-2.1
AT2G18060	*NAC037*	NAC	0.00	-2.6	0.00	-2.8	0.17	-1.6
AT4G36160	*NAC076*	NAC	0.00	-2.5	0.00	-2.7	1.00	-1.0
AT1G65910	*NAC028*	NAC	0.00	-2.1	0.00	-3.0	0.03	-1.6
AT4G28500	*NAC073*	NAC	0.21	-1.5	0.00	-2.6	0.00	-2.2
AT3G12910	*AT3G12910*	NAC	0.00	-∞	NA	NA	0.00	-4.9
AT1G62700	*NAC026*	NAC	0.00	-∞	0.00	-3.1	0.11	-1.7
AT5G64060	*NAC103*	NAC	0.02	-∞	0.00	-2.5	1.00	-1.1
AT2G44840	*ERF13*	ERF	0.35	1.6	0.01	-2.8	0.00	-24.5
AT2G46310	*CRF5*	ERF	0.00	-9.4	0.00	-5.0	0.02	-1.4
AT4G11140	*CRF1*	ERF	0.13	-2.1	0.00	-8.6	0.00	-2.7
AT2G25820	*ERF042*	ERF	NA	NA	0.00	-8.2	0.00	-2.4
AT4G27950	*CRF4*	ERF	0.05	-2.4	0.00	-3.1	0.01	-2.8
AT1G25470	*ERF116*	ERF	0.00	-2.1	0.00	-2.0	1.00	-1.1
AT5G13330	*ERF113*	ERF	0.00	-6.1	0.00	-∞	NA	NA
AT5G44210	*ERF9*	ERF	0.00	-2.7	0.00	-∞	1.00	1.2
AT1G22810	*ERF019*	ERF	0.00	-∞	NA	NA	0.00	-2.4
AT5G25810	*TINY*	ERF	0.00	-∞	0.00	-∞	1.00	1.0
AT5G65640	*BHLH93*	bHLH	0.00	-3.8	0.00	-3.6	1.00	1.0
AT1G12860	*SCRM2*	bHLH	0.00	-2.4	0.00	-4.5	0.00	-1.5
AT1G63650	*BHLH2*	bHLH	0.00	-3.0	0.00	-3.7	1.00	1.1
AT5G67110	*ALC*	bHLH	0.00	-3.0	0.00	-3.4	0.51	-1.3
AT2G46810	*BHLH70*	bHLH	0.00	-2.1	0.00	-4.3	0.00	-1.5
AT3G61950	*BHLH67*	bHLH	0.00	-2.7	0.00	-3.2	0.00	-1.7
AT4G36930	*SPT*	bHLH	0.00	-2.4	0.00	-2.9	0.10	-1.7
AT5G50915	*BHLH137*	bHLH	0.42	-1.3	0.00	-3.2	0.00	-2.0
AT5G51790	*BHLH120*	bHLH	NA	NA	0.00	-∞	0.00	-2.2
AT5G67060	*HEC1*	bHLH	0.00	-∞	0.00	-∞	NA	NA
AT3G52910	*GRF4_2*	GRF	0.00	-11.5	0.00	-16.9	0.09	-1.6
AT4G37740	*GRF2_2*	GRF	0.00	-2.8	0.00	-10.0	0.00	-1.8
AT2G22840	*GRF1_1*	GRF	0.00	-2.6	0.00	-5.1	1.00	1.1
AT2G45480	*AtGRF9*	GRF	0.00	-∞	0.00	-11.7	1.00	-1.1
AT2G06200	*GRF6_1*	GRF	0.02	-1.9	0.00	-3.0	0.00	-∞
AT2G36400	*GRF3_1*	GRF	0.00	-∞	0.00	-∞	0.72	-1.3
AT2G45050	*GATA2*	GATA	0.00	-5.6	0.00	-8.1	0.10	-1.7
AT3G16870	*GATA17*	GATA	0.00	-4.0	0.00	-3.4	0.00	-1.5

AT3G51080	*GATA6*	GATA	0.00	-2.2	0.00	-3.4	0.01	-1.4
AT3G06740	*GATA15*	GATA	0.00	-2.0	0.00	-2.7	0.00	-1.6
AT3G54810	*GATA8*	GATA	0.09	1.4	0.00	-2.2	0.00	-2.2
AT1G75240	*ZHD5*	ZF-HD	0.00	-25.7	0.00	-27.1	0.00	-1.8
AT1G14440	*ZHD4*	ZF-HD	0.00	-3.3	0.00	-13.9	0.00	-1.9
AT3G50890	*ZHD7*	ZF-HD	0.00	-5.2	0.00	-5.4	0.06	-1.5
AT5G65410	*ZHD1_2*	ZF-HD	0.00	-2.6	0.00	-4.3	0.00	-1.9
AT5G25475	*AT5G25475*	B3	0.00	-2.3	0.00	-6.8	0.13	-1.4
AT3G11580	*ARF32*	B3	0.00	-3.7	0.00	-4.3	0.23	1.6
AT2G36080	*ARF31*	B3	0.02	-2.6	0.00	-∞	0.46	1.6
AT5G42700	*AT5G42700*	B3	0.00	-∞	0.00	-∞	0.34	-∞
AT5G37260	*RVE2*	MYB_related	0.00	-5.9	0.00	-2.4	0.09	1.4
AT1G18330	*RVE7*	MYB_related	0.00	-2.7	0.00	-2.8	0.00	1.6
AT1G17460	*TRFL3*	MYB_related	0.00	-2.7	0.00	-2.3	0.26	-1.5
AT1G15720	*TRFL5*	MYB_related	0.00	-2.2	0.00	-2.4	0.11	-1.5
AT2G44910	*ATHB-4*	HD-ZIP	0.00	-11.4	0.00	-15.2	0.04	1.7
AT1G73360	*HDG11*	HD-ZIP	0.00	-4.3	0.00	-8.8	1.00	-1.1
AT3G60390	*HAT3*	HD-ZIP	0.00	-2.6	0.00	-3.1	0.00	1.6
AT3G22760	*TCX3*	CPP	0.00	-3.5	0.00	-10.8	0.01	-1.5
AT4G14770	*TCX2*	CPP	0.00	-3.4	0.00	-8.2	0.00	-1.8
AT2G20110	*TCX6*	CPP	0.00	-2.4	0.00	-3.7	0.41	-1.4
AT4G37750	*ANT*	AP2	0.00	-3.0	0.00	-6.0	0.00	-1.8
AT5G10510	*AIL6*	AP2	0.00	-2.5	0.00	-2.4	0.03	-1.7
AT5G57390	*AIL5*	AP2	0.00	-2.4	0.00	-2.2	0.01	-1.9
AT3G01330	*E2FF*	E2F/DP	0.00	-2.7	0.00	-5.5	0.57	-1.3
AT3G48160	*E2FE*	E2F/DP	0.00	-3.7	0.00	-4.0	0.01	-1.8
AT5G14960	*E2FD*	E2F/DP	0.00	-∞	0.00	-∞	0.11	-∞
AT3G19360	*AT3G19360*	C3H	0.00	-3.9	0.00	-4.1	0.00	-1.7
AT2G19810	*AT2G19810*	C3H	0.00	-5.0	0.00	-2.9	0.70	-1.1
AT2G28450	*AT2G28450*	C3H	0.00	-2.1	0.00	-2.4	0.00	-1.5
AT5G51910	*TCP19*	TCP	0.02	-2.8	0.00	-10.6	1.00	-1.0
AT2G37000	*TCP11*	TCP	0.00	-∞	0.00	-∞	NA	NA
AT3G50410	*DOF3.4*	Dof	0.01	-2.0	0.00	-5.1	0.21	-1.5
AT3G61850	*DOF3.7*	Dof	0.00	-2.1	0.00	-3.1	0.00	-1.9
AT1G68120	*BPC3*	BBR-BPC	0.00	-2.3	0.00	-4.5	1.00	1.1
AT2G01930	*BPC1*	BBR-BPC	0.01	-2.5	0.00	-2.5	0.42	1.2
AT1G63100	*SCL28_1*	GRAS	0.00	-4.3	0.00	-15.4	0.07	-1.6
AT1G46264	*HSFB4*	HSF	0.00	-2.5	0.00	-8.9	0.10	-1.5
AT3G18010	*WOX1*	WOX	0.00	-6.5	0.00	-4.1	0.08	-1.9
AT3G50700	*IDD2*	C2H2	0.00	-3.4	0.00	-3.5	1.00	1.0
AT4G39070	*BBX20*	DBB	0.04	-3.3	0.04	-3.5	0.60	-1.3
AT1G68840	*RAV2*	RAV	1.00	-1.1	0.03	-4.0	0.00	-2.5
AT1G19850	*ARF5*	ARF	0.00	-2.8	0.00	-3.5	0.15	-1.3
AT5G15840	*CO*	CO-like	0.00	-3.6	0.00	-2.4	0.70	-1.4
AT2G47260	*WRKY23*	WRKY	0.00	-2.8	0.00	-2.9	0.00	-1.9
AT2G02060	*AT2G02060*	G2-like	0.00	-2.5	0.00	-3.0	0.06	1.6
AT1G61730	*AT1G61730*	GeBP	0.00	-2.1	0.00	-2.3	0.00	-1.5
AT1G23380	*KNAT6*	TALE	0.01	-∞	0.03	-∞	0.18	-1.7
AT1G47760	*AGL102*	M-type_MADS	0.03	-∞	0.00	-∞	0.99	-1.4
AT1G31310	*AT1G31310*	Trihelix	0.00	-∞	0.00	-∞	NA	NA

[a, b] Gene locus and symbols were retrieved from TAIR10.
[c] Transcription factors were classified into families based on the assignment rules in PlantTFDB. Within each family, transcription factors were ranked according to highest individual fold-change.
[d] *P*-value adjusted by FDR, calculated by *Empirical analysis of DGE* in CLC Genomics Workbench.
[e] FC - fold-change in expression of genes between data sets (comparing samples with abundant vs no wall ingrowths), calculated by *Empirical analysis of DGE* in CLC Genomics Workbench.

[f] NA indicates gene that were identified as not expressed in both samples from each experimental comparison thus not available fold-change or *P*-value.
[g] Fold-changes ≤-2 or -∞ with $P < 0.05$ are indicated in red.
[h] -∞ indicates gene was not expressed (exon counts <10) in samples with abundant wall ingrowth deposition, but was expressed (exon counts ≥ 10) in samples with no detectable wall ingrowths.

Supplementary Table 4. Details of T-DNA insertional mutants for selected NAC-III-2 and III-3 transcription factors identified as putative candidate regulators of wall ingrowth deposition in Arabidopsis PP TC development.

Gene	Mutant allele	Mutant name	Resources	Publication
NAC032	*GK_852A11*	*nac002/032*	Crossed by GABI-DUPLO project and donated to NASC	Bolle et al., 2013
NAC002	*GK_239B09*			
NAC055	*SALK_014331*	*nac019/055/072*	Dong Lab, Duke University, US	Zheng et al., 2012
NAC019	*SALK_096295*			
NAC072	*SALK_083756*			
NAC056	*SM_3_28017*	*nac056/018*	Hara-Nishimura Lab, Kyoto University, Japan	Kunieda et al., 2008
NAC018	*WiscDsLox364F11*			
